# DNASE1L3-mediated PANoptosis enhances the efficacy of combination therapy for advanced hepatocellular carcinoma

**DOI:** 10.7150/thno.102995

**Published:** 2024-10-14

**Authors:** Jingchun Wang, Yu Chen, Yanquan Xu, Jiangang Zhang, Shuai Yang, Yu Zhou, Juan Lei, Ran Ren, Yang Chen, Huakan Zhao, Yongsheng Li, Shiming Yang

**Affiliations:** 1 Department of Gastroenterology, Second Affiliated Hospital, Army Medical University; Chongqing 400037, China.; 2 Department of Medical Oncology, Chongqing University Cancer Hospital; Chongqing 400030, China.; 3 Clinical Medicine Research Center, Second Affiliated Hospital, Army Medical University; Chongqing 400037, China.; 4 Department of Pathology, First Affiliated Hospital, Army Medical University; Chongqing 400037, China.

**Keywords:** advanced hepatocellular carcinoma, combination therapy, DNASE1L3, PANoptosis

## Abstract

**Rationale:** The introduction of combination therapy utilizing tyrosine kinase inhibitors (TKIs) and immune checkpoint inhibitors for advanced hepatocellular carcinoma (HCC) has significantly altered the management of affected patients. However, the absence of predictive biomarkers to identify those who would derive the greatest benefit from this combination therapy underscores the necessity for further enhancements in its efficacy.

**Methods:** In this study, we performed a proteomic analysis on surgical specimens from patients who either responded to or did not respond to combination therapy with sorafenib and programmed death-1 (PD-1) monoclonal antibody (mAb). We employed *in vitro* experiments, including immunocytochemistry, co-immunoprecipitation, and transmission electron microscopy, to elucidate the mechanism of DNASE1L3-induced PANoptosis. Additionally, we assessed the function of DNASE1L3 in combination therapy using a mouse liver orthotopic tumor model and clinical samples.

**Results:** Our findings indicated that the levels of deoxyribonuclease 1 like 3 (DNASE1L3) were significantly elevated in the cohort of patients who responded to treatment, correlating with the sorafenib-induced programmed cell death (PCD) of HCC cells. Further experimentation revealed that DNASE1L3 facilitated the generation of double-strand deoxyribonucleic acid (dsDNA) breaks and activated the absent in melanoma 2 (AIM2) pathway during sorafenib-induced HCC cell death, ultimately culminating in PANoptosis. Moreover, DNASE1L3-induced PANoptosis augmented the activation of anti-tumor immunity within the tumor microenvironment (TME), thereby enhancing the efficacy of the combination therapy involving sorafenib and PD-1 mAb.

**Conclusion:** Our findings offer valuable insights into the mechanisms underlying DNASE1L3's role in sorafenib sensitivity and position DNASE1L3 as a promising predictive biomarker and target for improving outcomes in combination therapy for HCC.

## Introduction

The treatment of hepatocellular carcinoma (HCC) has recently evolved to incorporate targeted therapy alongside immunotherapy 1, 2. Sorafenib, a well-known tyrosine kinase inhibitor (TKI), has served as a first-line treatment for advanced HCC for over a decade 3. In recent years, the combination of sorafenib with programmed death-1 (PD-1) monoclonal antibody (mAb) immunotherapy has gained widespread clinical application, resulting in enhanced efficacy of immunotherapy and improved patient outcomes 4. Nevertheless, certain patients continue to exhibit inherent resistance to this combination therapy 5, 6. Therefore, it is essential to address the pressing challenge of increasing the efficacy of this treatment.

Sorafenib has been shown to inhibit tumor angiogenesis by targeting vascular endothelial growth factor receptor (VEGFR) 7. Additionally, it directly induces programmed cell death (PCD) in tumor cells 8. One specific form of PCD, termed PANoptosis, has emerged as an immunogenic process exhibiting characteristics akin to pyroptosis, apoptosis, and necroptosis 9. In recent years, the role of PANoptosis in tumors has garnered increasing attention 10. Bioinformatics studies indicate that HCC tissues with elevated expression of PANoptosis-related genes demonstrate a more robust response to immunotherapy and are enriched in pathways associated with DNA damage, drug metabolism, cytokines, and immune receptors 11. Consequently, enhancing the immunogenic PCD of tumor cells presents a promising strategy for improving the effectiveness of combination therapy.

Deoxyribonuclease 1 like 3 (DNASE1L3) belongs to the deoxyribonuclease 1 family, which is responsible for cleaving nuclear chromosomal DNA in an internucleosomal manner, independent of proteolytic assistance 12-15. This enzymatic activity generates nucleosome-sized DNA fragments and double-strand DNA (dsDNA) breaks during apoptosis 14. However, the precise role of DNASE1L3 in other forms of cell death remains to be elucidated. In recent years, the involvement of nucleases in tumor biology has garnered considerable attention. Previous studies have shown that DNASE1L3 expression is significantly downregulated in various tumors 16, 17, with particularly lower expression levels detected in HCC tissues compared to adjacent normal tissues. Furthermore, DNASE1L3 has been demonstrated to inhibit HCC cell proliferation by reducing glycolysis and promoting apoptosis 18. Additionally, DNASE1L3 contributes to tumor immunity; bioinformatic analyses reveal a positive correlation between DNASE1L3 and the HCC immune signature, as well as immune cell infiltration within the tumor microenvironment (TME) 19. Moreover, the inhibition of DNASE1L3 disrupts the formation of apoptosis-associated speck-like protein (ASC) and the release of inflammatory factors 20. Nonetheless, further experimental validation and exploration of the underlying mechanisms are essential. There is also a correlation between DNASE1L3 expression and treatment outcomes in HCC; higher levels of DNASE1L3 are associated with improved overall survival following radical resection 21. Besides, DNASE1L3 enhances the sensitivity of HCC cells to etoposide and sorafenib, although the mechanisms underlying this effect remain unclear 22, 23. Therefore, elucidating the function and mechanisms of DNASE1L3 in HCC treatment and TME immunity is of paramount importance.

In this study, we employed proteomic analysis to examine surgical specimens from HCC patients who either responded or did not respond to treatment with sorafenib in combination with a PD-1 mAb. Our results indicate that DNASE1L3 may represent a promising target for enhancing the efficacy of this combination therapy in HCC.

## Results

### DNASE1L3 expression correlates with improved survival and treatment response in HCC

**Figure 1:**
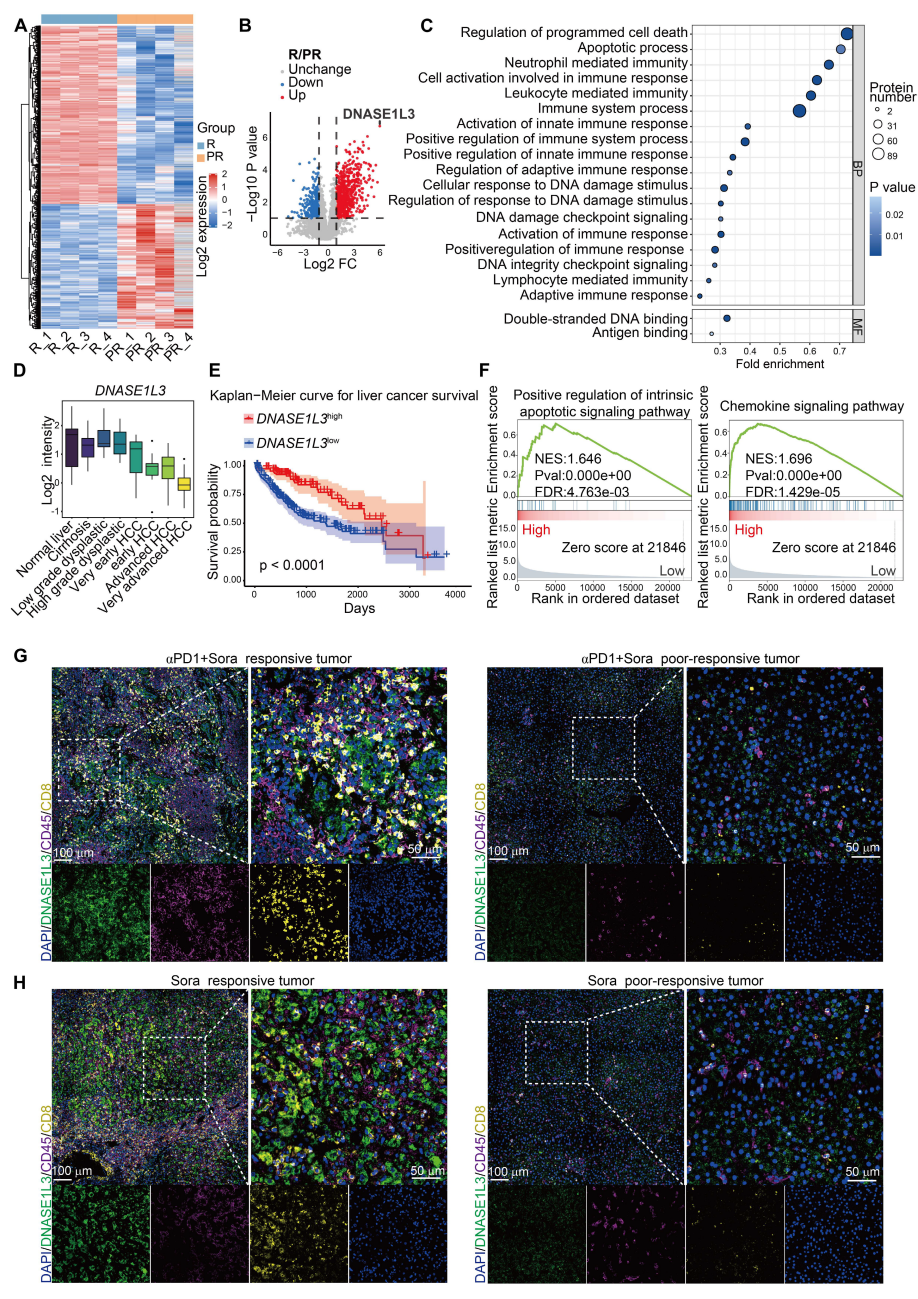
** DNASE1L3 expression in HCC tissue of combination therapy with sorafenib and PD-1 mAb.** (**A**) Heatmaps showing the expression of DEPs between responsive tumor group (R) and poor-responsive tumor group (PR). (**B**) Volcano plot of the -Log10 p value against the Log2 [fold-change (Log2 FC) of the DEPs. Dots are colored by logFC. Vertical dotted lines indicate |log2 FC| = 2, and horizontal dotted line indicates adjusted p value = 0.05. (**C**) GO enrichment up analysis of DEPs between responsive tumor group and poor-responsive tumor group. (**D**) Log2 median centered intensity of *DNASE1L3* in different stages of HCC from the Wurmbach liver dataset. (**E**) Kaplan-Meier survival curve based on the relative expression of *DNASE1L3* in liver cancer tissue from the TCGA database. (**F**) Representative GSEA of differentially expressed genes (DEGs) from liver cancer tissues with high or low expression of *DNASE1L3* in the TCGA database. (**G** and **H**) IF assays were used to verify the expression of DAPI (blue), DNASE1L3 (green), CD45 (purple) and CD8 (yellow) for the HCC surgical specimens from responsive and poor-responsive patients to sorafenib monotherapy and combination therapy. αPD-1 represents PD-1 mAb. Scale bars in overall images (left) is 100 μm, and in enlarged images (right) is 50 μm. Images with separated channels are below.

To investigate the relationship between tumor biomarkers and the prognosis of combination therapy involving sorafenib and PD-1 mAb, we performed a proteomics analysis on surgical specimens obtained from eight patients who received this treatment. These patients were categorized into two groups: the clinically responsive group and the poor-responsive group, based on the Response Evaluation Criteria in Solid Tumors Version (RECIST) 1.1 criteria 24 (Figure S1A). Our analysis of differentially expressed proteins revealed that 1031 proteins were significantly upregulated in the treatment-responsive group (Figure 1A and Figure S1B), with DNASE1L3 identified as one of the most notably upregulated proteins (Figure 1B). DNASE1L3 is actively involved in cell death by degrading DNA to produce nucleosome-sized dsDNA. Furthermore, Gene Ontology (GO) analysis indicated a significant enrichment in pathways associated with PCD, DNA damage and binding, and immune cell activation in the responsive group (Figure 1C). Given these findings, we are keen to further explore the potential positive role of DNASE1L3 in enhancing the efficacy of sorafenib in combination with PD-1 mAb.

The expression of DNASE1L3 has been reported to be significantly downregulated in various tumors 16. In this study, we investigated DNASE1L3 protein expression in 12 pairs of cancerous and adjacent non-cancerous tissues from HCC patients. Our findings demonstrated that DNASE1L3 expression was significantly lower in cancer tissues compared to adjacent non-cancerous tissues (Figure S1C).

Further analysis of The Cancer Genome Atlas (TCGA) database revealed that *DNASE1L3* is among the most downregulated genes in HCC (Figure S1D), with its expression progressively decreasing as HCC stages advance (Figure 1D). To elucidate the role of DNASE1L3 in HCC, we categorized RNA-seq raw data from 424 liver hepatocellular carcinoma (LIHC) samples obtained from the TCGA database into two groups: the top 100 samples with high *DNASE1L3* expression and the top 100 samples with low *DNASE1L3* expression (Figure S1E-G). Our results indicated that HCC patients with high *DNASE1L3* expression experienced significantly longer survival compared to those with low* DNASE1L3* expression levels (Figure 1E). Additionally, we observed upregulations of apoptosis and immune-related pathways in patients exhibiting high *DNASE1L3* expression (Figure 1F). Furthermore, our analysis of the liver cancer database suggests that higher expression of *DNASE1L3* is positively correlated with better prognosis following both sorafenib therapy and immunotherapy (Figure S1H, I). These findings are corroborated by proteomic data, indicating that DNASE1L3 may play a crucial role in enhancing the prognosis of HCC treatment.

Previous studies have indicated the mechanisms of PCD induced by sorafenib in HCC cells, including apoptosis and ferroptosis, 25, 26. To further investigate the role of DNASE1L3 in the context of sorafenib monotherapy, we collected eight surgical specimens: four from patients who exhibited a positive response to sorafenib and four from those who did not respond. Immunofluorescence (IF) staining revealed that DNASE1L3 expression levels in HCC cells were significantly higher in both the sorafenib monotherapy and combination therapy responding groups compared to the poor-responding group. Furthermore, the proportion of CD8^+^ T cells was also elevated in the responding group (Figure 1G, H and S1J, K). These results suggest that DNASE1L3 expression in HCC cells may correlate with the efficacy of sorafenib treatment and may exert a beneficial influence on the TME.

### DNASE1L3 enhances the sensitivity of HCC cells to sorafenib-induced cell death

**Figure 2:**
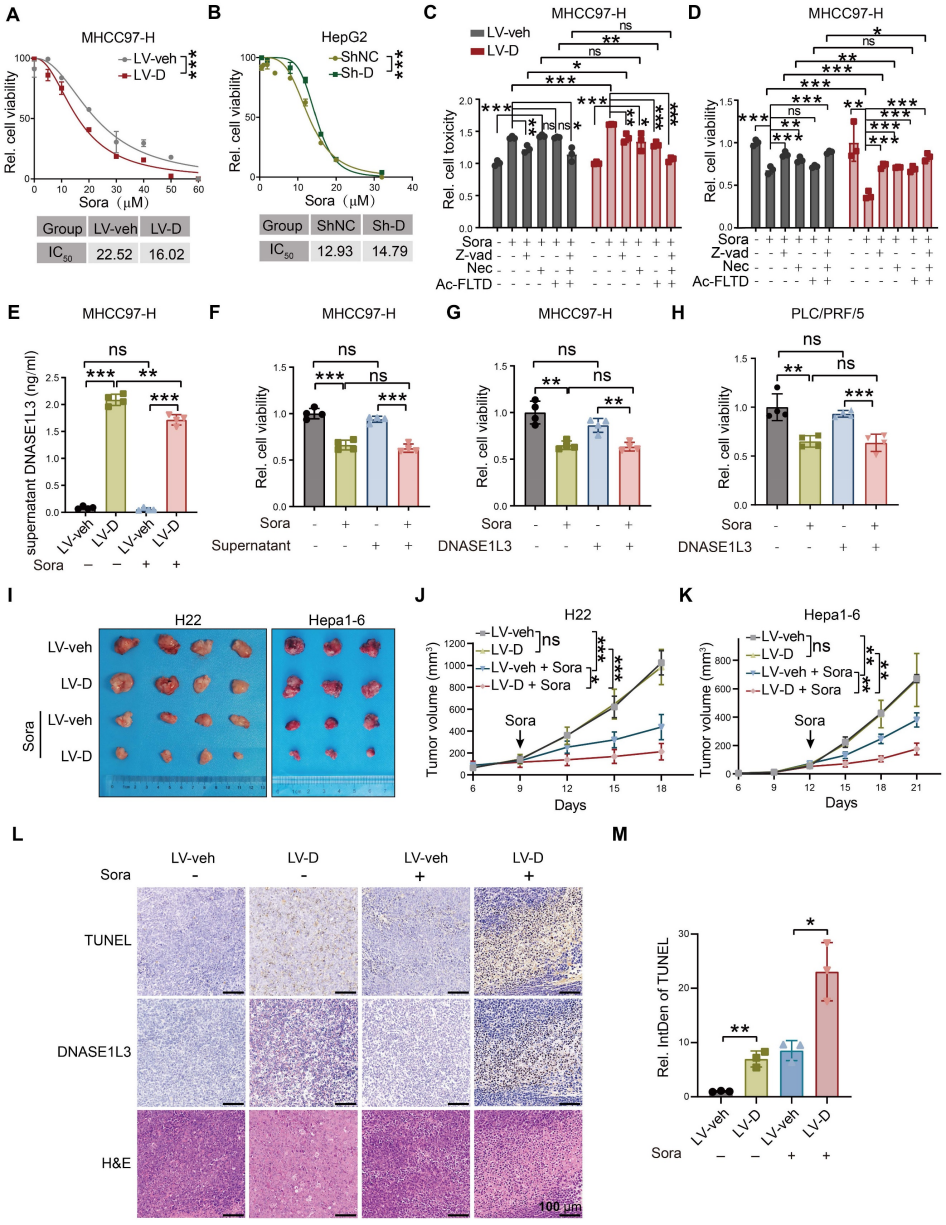
** DNASE1L3 enhances the sensitivity of HCC cells to sorafenib.** (**A** and **B**) CCK8 sensitivity analysis with sorafenib concentration gradient from 0 μM to 60 μM in LV-veh, LV-D MHCC97-H HCC cells (**A**) and sorafenib concentration gradient from 0 μM to 40 μM in ShNC, Sh-D HepG2 HCC cells (**B**). IC_50_ was calculated below. (**C** and **D**) Cell toxicity (**C**) and viability (**D**) assessments of LV-veh, LV-D MHCC97-H HCC cells treated or not treated with 20 μM sorafenib for 48 h in combination with the apoptosis inhibitor Z-VAD-FMK (VAD, 25 μM), the necroptosis inhibitor necrostatin (Nec, 20 μM), and the pyroptosis inhibitors Ac-FLTD (20 μM). (**E**) DNASE1L3 level in MHCC97-H cells culture supernatant was detected though ELISA assay. The sorafenib group were treated with 20 µM sorafenib for 48 h. (**F**) The supernatant of LV-D MHCC97-H cells was collected after 48 h incubation and used to incubate MHCC97-H cells with sorafenib (20 µM) for 48 h. The relative cell viability was detected with CCK-8 assay. (**G** and **H**) MHCC97-H (**G**) and PLC/PRF/5 cells (**H**) were incubated with DNASE1L3 recombinant protein (2 ng/mL) and sorafenib (20 µM) for 48 h. The relative cell viability was detected with CCK-8 assay. (**I**) Photographs of the excised tumors from nude mice injected LV-veh, LV-D H22 and Hepa1-6 mouse liver cancer cells subcutaneously. (**J** and **K**) Statistical analysis of the tumor volumes (mm^3^) of H22 model (**J**) and Hepa1-6 model (**K**). Sorafenib treatment was applied beginning at the time pointed by the arrow. (**L**) Representative H&E and IHC staining images (left panel) of DNASE1L3 and TUNEL in the Hepa1-6 subcutaneous tumor in nude mice with or without sorafenib. Scale bar = 100 μm. (**M**) Quantification of the apoptotic index (TUNEL staining) was on the right panel. IntDen is the abbreviation of integrated density. The data in (**A-D**, **K, M**) are representative of three independent experiments, while the data in (**E-H, J**) are representative of four independent experiments and (**E**-**H)** have five replication wells. All the data are presented as mean ± SD. *p < 0.05, **p < 0.01, ***p < 0.001, ns represents no significant difference.

To investigate the specific role of DNASE1L3 in the treatment of HCC, we overexpressed DNASE1L3 (LV-D) in two HCC cell lines with low expression levels, namely MHCC97-H and PLC/PRF/5. Additionally, we knocked down DNASE1L3 (Sh-D) in HepG2 cells (Figure S2A-G). Our findings indicated that the modulation of DNASE1L3 expression, through both overexpression and knockdown, did not affect the proliferation rates of HCC cells (Figure S2H-K). However, we observed that overexpression of DNASE1L3 significantly decreased the half-maximal inhibitory concentration (IC_50_) of sorafenib in HCC cells (Figure 2A and Figure S2L). Conversely, the knockdown of DNASE1L3 resulted in a slight increase in the IC_50_ of sorafenib treatment (Figure 2B), suggesting that DNASE1L3 may enhance the sensitivity of HCC cells to sorafenib. To further elucidate the type of cell death induced by sorafenib in relation to DNASE1L3, we employed specific inhibitors targeting various cell death pathways. Notably, the apoptosis inhibitor Z-VAD-FMK partially inhibited sorafenib-induced cell death in the control group. In LV-D HCC cells, the induction of cell death by sorafenib was also partially inhibited by Z-VAD-FMK, as well as by the necroptosis inhibitor necrostatin and the pyroptosis inhibitor ac-FLTD-CMK. Moreover, the combination of these three inhibitors significantly reduced sorafenib-induced cell death (Figure 2C and S2M). Cell viability assays indicated that the survival of control PLC/PRF/5 HCC cells (LV-veh) after sorafenib treatment was only partially restored by Z-VAD-FMK. In control MHCC97-H HCC cells, necrostatin also led to a modest improvement in survival post-sorafenib treatment, although its effect was less pronounced than that of Z-VAD (Figure 2D). In contrast, LV-D HCC cells exhibited a partial increase in cell survival following sorafenib treatment with all three death inhibitors, and their combination significantly enhanced cell viability (Figures 2D and S2N). Based on these results, we hypothesized that DNASE1L3 enhance the sensitivity of HCC cells to sorafenib by promoting multiple forms of cell death, collectively referred to as PANoptosis.

Given that DNASE1L3 is a secreted protein with enzymatic activity both intracellularly and extracellularly, we investigated the levels of DNASE1L3 protein in the culture supernatants of LV-D and LV-veh cells. Our findings revealed that the DNASE1L3 protein level in the culture supernatant of LV-D cells was significantly higher than that in LV-veh cells. Furthermore, we observed a down-regulation of DNASE1L3 secretion in LV-D cells following treatment with sorafenib compared to the untreated condition (Figure 2E).

Notably, culturing LV-veh cells with LV-D cell culture supernatants did not affect their sensitivity to sorafenib (Figure 2F). Moreover, the introduction of recombinant DNASE1L3 protein into HCC cell culture supernatants had no influence on the cell death induced by sorafenib (Figure 2G, H). These results suggest that DNASE1L3 enhances sorafenib-induced cell death primarily dependent on its intracellular functions. Next, we overexpressed DNASE1L3 in mouse liver cancer cells H22 and Hepa1-6 (Figure S2O-R), and injected them subcutaneously into nude mice. The overexpression of DNASE1L3 did not significantly affect the growth of subcutaneous tumors. However, after treatment with sorafenib, the subcutaneous tumors in the LV-D group were significantly smaller compared to those in the LV-veh group (Figure 2I-K). Moreover, the TUNEL assay results indicated a significant increase in dsDNA breaks in the LV-D group compared to the LV-veh group (Figure 2L, M). These findings suggest that DNASE1L3 plays a critical role in enhancing the sensitivity of HCC to sorafenib.

### DNASE1L3 promotes sorafenib-induced PANoptosis in HCC cells

**Figure 3:**
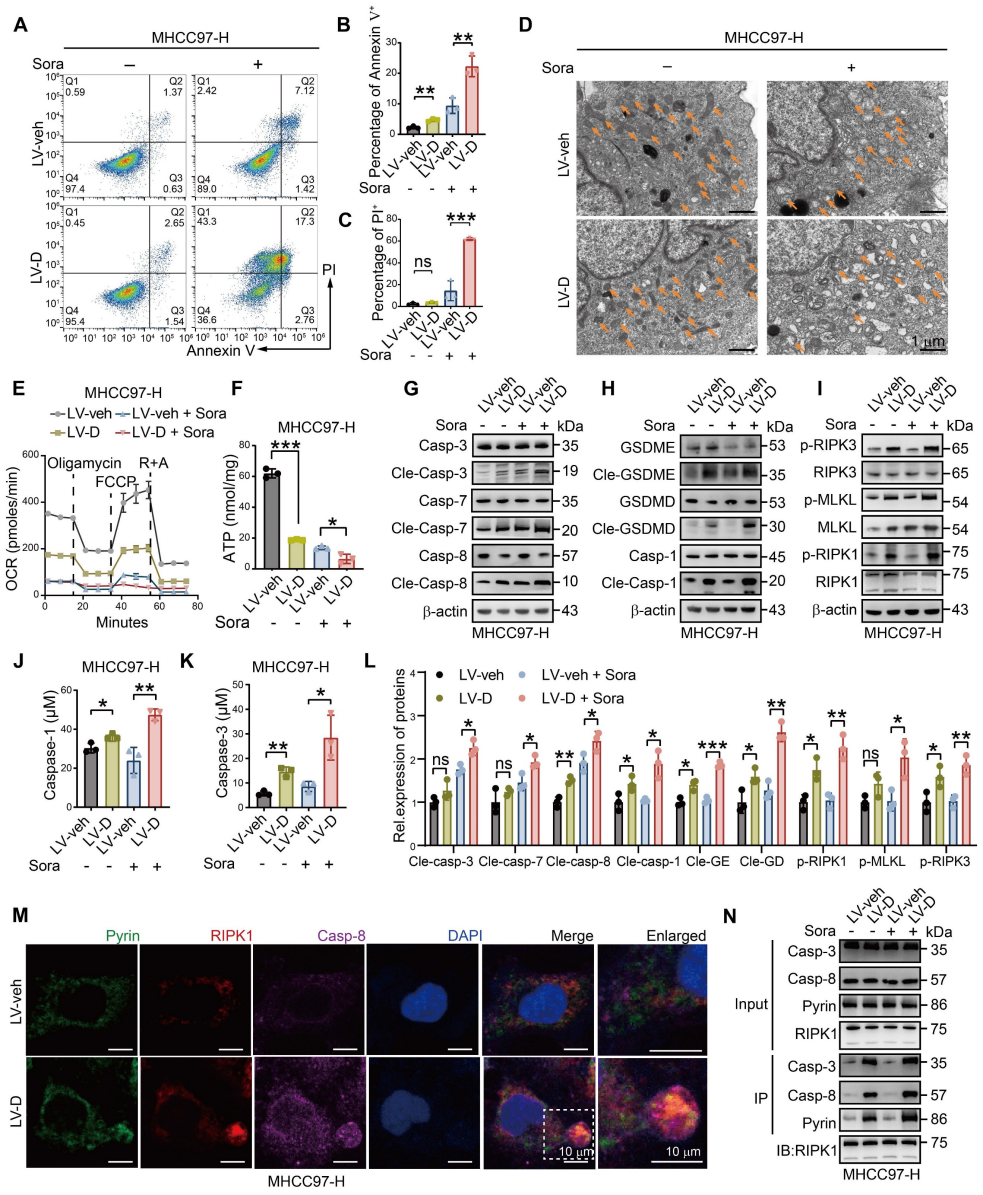
**DNASE1L3 facilitates sorafenib-induced PANoptosis in HCC cells.** (**A-C**) FCM (**A**) and quantification analysis (**B** and **C**) with Annexin V/PI staining evaluating the percentages of live cells among the LV-veh and LV-D MHCC97-H HCC cells with or without sorafenib (20 μM, 48 h). (**D**) Electron microscope images of LV-veh and LV-D MHCC97-H HCC cells treated with or without sorafenib (20 μM, 24 h). Mitochondria were represented by orange arrows. Scale bar = 1 μm. (**E**) Seahorse assay detected OCR after drug supplement including oligomycin, carbonyl cyanide 4-(trifluoromethoxy)phenylhydrazone (FCCP), rotenone (R) and antimycin A (A). (**F**) ATP detection in LV-veh and LV-D MHCC97-H HCC cells treated with sorafenib (20 μM, 24 h). (**G**-**I**) Western blotting analysis of apoptosis-related proteins (**G**), pyroptosis-related proteins (**H**), and necroptosis-related proteins (**I**). (**J** and **K**) Enzyme activities of caspase-1 (**J**) and caspase-3 (**K**) in LV-veh and LV-D MHCC97-H HCC cells treated with sorafenib (20 μM, 24 h). (**L**) Quantification of the western blotting analysis. (**M**) IF assay represented Pyrin (green), RIPK1 (red), caspase-8 (purple) and DAPI (blue) in LV-veh and LV-D MHCC97-H HCC cells treated with sorafenib (20 μM, 24 h). The co-expression assembled spot is PANoptosome. Scale bar = 10 μm. (**N**) Co-IP assay showed the combination of Pyrin, RIPK1, caspase-3 and caspase-8 in LV-veh and LV-D MHCC97-H HCC cells with or without sorafenib (20 μM, 24 h). The data in (**B**, **C**, **E**, **F**, **J**, **K**, **L**) are representative of three independent experiments. All the data are presented as mean ± SD. *p < 0.05, **p < 0.01, ***p < 0.001, ns represents no significant difference.

To investigate whether DNASE1L3 promotes sorafenib-induced death *via* the PANoptosis pathway in HCC cells, we conducted a series of experiments. Firstly, we observed that the level of apoptosis in LV-D HCC cells was significantly higher compared to LV-veh HCC cells following sorafenib treatment (Figure 3A-C and Figure S4A). In addition, LV-D HCC cells displayed more pronounced mitochondrial damage, characterized by mitochondrial vacuolization, cristae breakage, a reduced number of mitochondria, and decreased membrane potential, after sorafenib treatment (Figure 3D, S3A-C, and S4B). The Seahorse assay and adenosine triphosphate (ATP) assay revealed that the mitochondrial oxygen consumption rate (OCR) and ATP production levels were significantly lower in LV-D HCC cells compared to the LV-veh group after sorafenib treatment (Figure 3E, F, and S3D-F). Moreover, the expressions of apoptosis, pyroptosis, and necroptosis-related proteins were remarkably upregulated in LV-D HCC cells, while they were slightly downregulated in Sh-D HCC cells following sorafenib treatment (Figure 3G-I, L and S3G-I, L). The enzyme activities of caspase-1, a crucial protein in the pyroptosis pathway, and caspase-3, an essential protein in the apoptosis pathway, were significantly higher in LV-D HCC cells compared to LV-veh HCC cells after sorafenib treatment (Figure 3J, K). Furthermore, we assessed the levels of mitochondrial lipid reactive oxygen species (ROS) following sorafenib treatment and observed that lipid ROS levels were marginally elevated in LV-veh HCC cells compared to LV-D HCC cells (Figure S3J, K). Given that lipid ROS is crucial for ferroptosis 27, these findings suggest that ferroptosis may not play a role in DNASE1L3-induced PANoptosis.

The assembly of the PANoptosome is a critical process during the early stages of PANoptosis. In our study, we identified the presence of the PANoptosome in sorafenib-treated LV-D HCC cells, which was composed of essential PANoptotic proteins, including Pyrin, receptor-interacting protein kinase 1 (RIPK1), and caspase-8 (Figure 3M and S4C). Co-immunoprecipitation (Co-IP) experiments further demonstrated the assembly of these key proteins in LV-D HCC cells, revealing an increased interaction following sorafenib induction (Figure 3N). Subsequently, we assessed cell morphology using electron microscopy at various time points after sorafenib treatment. At 48 h, LV-D HCC cells exhibited characteristics of inflammatory cell death, such as cell membrane rupture, cytoplasmic vacuolization, organelle disruption, and chromosome condensation. In contrast, LV-veh HCC cells displayed features of apoptotic cell death, including membrane budding, the formation of apoptotic vesicles, and nuclear condensation (Figure S3M and S5D). These findings suggest that LV-D HCC cells are more prone to undergo inflammatory PCD, known as PANoptosis, upon sorafenib induction, whereas LV-veh HCC cells are more likely to undergo apoptosis.

### DNASE1L3 mediates PANoptosis through its deoxyribonuclease activity

The function of DNASE1L3 was initially thought to be linked to its deoxyribonuclease activity 28. Consequently, we examined the expression of phosphorylated H2AX histone protein (γH2AX), which serves as a marker for dsDNA breaks. Our analysis revealed that γH2AX expression was elevated in LV-D HCC cells compared to LV-veh HCC cells. Following sorafenib induction, γH2AX levels in LV-D HCC cells increased rapidly within 24 h, followed by a gradual decline.

**Figure 4:**
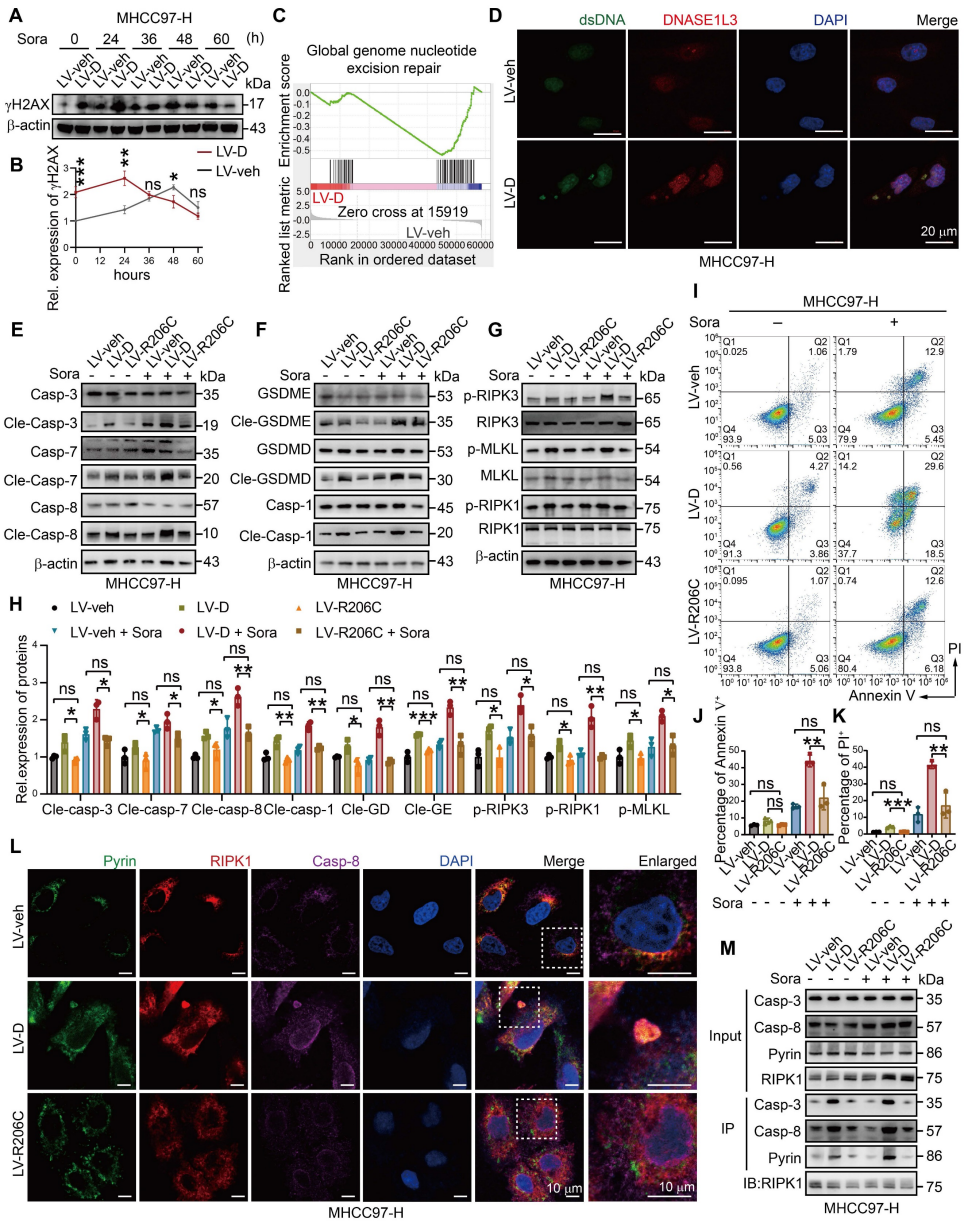
**DNASE1L3 promotes sorafenib-induced PANoptosis of HCC cells.** (**A** and **B**) The time-dependent expression of γH2AX in LV-veh and LV-D MHCC97-H HCC cells after sorafenib (20 μM) (**A**) was quantified (**B**). (**C**) Representative GSEA in RNA-seq from LV-veh and LV-D MHCC97-H HCC cells. (**D**) IF staining of dsDNA (green), DNASE1L3 (red) and DAPI (blue) in LV-veh and LV-D MHCC97-H HCC cells treated with sorafenib (20 μM, 24 h). Scale bar = 20 μm. (**E**-**H**) Western blotting analysis of apoptosis-related proteins (**E**), pyroptosis-related proteins (**F**), necroptosis-related proteins (**G**) in LV-veh, LV-D and LV-R206C MHCC97-H HCC cells treated with sorafenib (20 μM, 24 h), and the quantification of western blotting (**H**). (**I**-**K**) FCM (**I**) and quantification analysis (**J** and **K**) with Annexin V/PI staining evaluating the percentages of positive cells among the LV-veh, LV-D and LV-R206C MHCC97-H HCC cells with or without sorafenib (20 μM, 48 h). (**L**) IF assay represented Pyrin (green), RIPK1 (red), caspase-8 (purple) and DAPI (blue) in LV-veh, LV-D and LV-R206C MHCC97-H HCC cells treated with sorafenib (20 μM, 24 h). The co-expression assembled spot is PANoptosome. Scale bar = 10 μm. (**M**) Co-IP assay of Pyrin, RIPK1, caspase-3 and caspase-8 in LV-veh, LV-D and LV-R206C MHCC97-H HCC cells treated with or without sorafenib (20 μM, 24 h). The data in (**B**, **H**, **J**, **K**) are representative of three independent experiments. All the data are presented as mean ± SD. *p < 0.05, **p < 0.01, ***p < 0.001, ns represents no significant difference.

In contrast, LV-veh HCC cells exhibited a slower increase in γH2AX expression, which then decreased sharply after 48 h (Figure 4A, B). Electron microscopy images and observed pathological features, such as mitochondrial swelling in LV-D HCC cells at 24 h, suggest that significant PANoptosis occurred at 48 h. Conversely, LV-veh HCC cells displayed considerable apoptosis at 60 h. Therefore, we hypothesize that DNASE1L3 actively cleaves chromatin DNA into nucleosome-sized fragments following sorafenib induction, rendering the DNA incapable of effective repair and leading to cell death. To further verify this hypothesis, RNA-seq analysis was conducted on sorafenib-treated LV-D and LV-veh MHCC97-H HCC cells, revealing a significant inhibition of the cellular nucleosome repair pathway upon DNASE1L3 overexpression (Figure 4C). Additionally, dsDNA staining demonstrated substantial dsDNA aggregation in the cytoplasm of LV-D HCC cells after sorafenib treatment, further corroborating our hypothesis (Figure 4D).

To confirm the role of DNASE1L3 in promoting the initiation of PANoptosis through its deoxyribonuclease activity, we created a mutant DNASE1L3 protein with inactivated deoxyribonuclease function. The deoxyribonuclease activity of DNASE1L3 is contingent upon its enzymatic activity domain, located within amino acids 194-231. Previous studies have identified a mutant DNASE1L3 protein in the population, characterized by a mutation at position 206, where arginine (R) is substituted with cysteine (C). This alteration results in the loss of deoxyribonuclease activity and is associated with the development of autoimmune diseases, such as lupus erythematosus 29, 30 (Figure S5A). We overexpressed the DNASE1L3 protein containing the mutated amino acid (LV-R206C) in HCC cells and observed a significantly lower expression of γH2AX compared to LV-D HCC cells, which was more akin to the expression levels seen in LV-veh HCC cells (Figure S5B, C). This finding suggests a loss of deoxyribonuclease activity in the mutant DNASE1L3 protein. Furthermore, we noted that PANoptosis-related proteins were drastically upregulated in LV-D HCC cells, while no significant differences were observed between LV-R206C and LV-veh HCC cells (Figure 4E-H and S5D-G). Additionally, we found no accumulation of dsDNA in LV-veh and LV-R206C HCC cells (Figure S5H). LV-R206C HCC cells also displayed significantly reduced levels of apoptosis and mitochondrial damage compared to LV-D cells, aligning more closely with the characteristics of LV-veh cells (Figure 4I-K and S5I). Moreover, we did not detect significant formation of PANoptosome in LV-R206C HCC cells (Figure 4L). Co-IP assays further supported these findings, demonstrating a markedly reduced interaction among PANoptosis-related proteins in LV-R206C cells compared to LV-D HCC cells (Figure 4M). Collectively, these results indicate that the absence of deoxyribonuclease function in DNASE1L3 is ineffective in inducing PANoptosis in HCC cells.

### DNASE1L3 mediates PANoptosis through activating the AIM2 pathway

**Figure 5:**
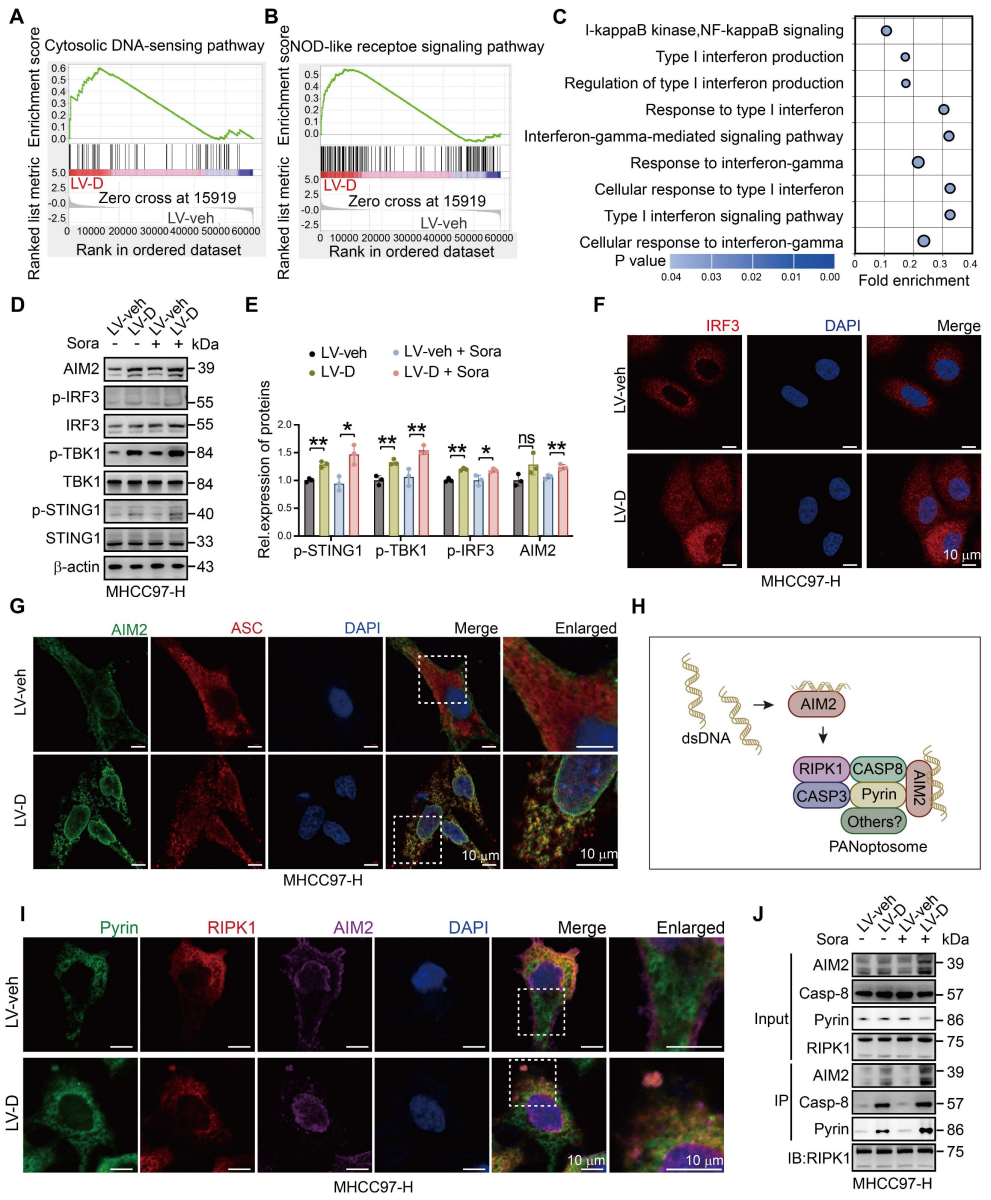
** DNASE1L3 activates AIM2 and cGAS-STING1 pathway.** (**A** and **B**) Representative GSEA in RNA-seq from LV-veh and LV-D MHCC97-H HCC cells with sorafenib (20 μM, 24 h). (**C**) GO enrichment analysis of upregulated DEGs between LV-veh and LV-D MHCC97-H HCC cells. (**D** and **E**) Western blotting analysis of cGAS-STING1 and AIM2 pathway-related proteins in LV-veh and LV-D MHCC97-H HCC cells with sorafenib (20 μM, 24 h). (**F**) IF staining of IRF3 (red) and DAPI (blue) in LV-veh and LV-D MHCC97-H HCC cells with sorafenib (20 μM, 24 h). Scale bar = 10 μm. (**G**) IF staining of expression of AIM2 (green), ASC (red) and DAPI (blue) in LV-veh and LV-D MHCC97-H HCC cells with sorafenib (20 μM, 24 h). Scale bar = 10 μm. (**H**) The diagrammatic drawing of AIM2-related PANoptosome formation. (**I**) IF assay of Pyrin (green), RIPK1 (red), AIM2 (purple) and DAPI (blue) in LV-veh and LV-D MHCC97-H HCC cells treated with sorafenib (20 μM, 24 h). The co-expression assembled spot represents PANoptosome. Scale bar = 10 μm. (**J**) Co-IP assay showed the combination of Pyrin, RIPK1, AIM2 and caspase-8 in LV-veh and LV-D MHCC97-H HCC cells with or without sorafenib (20 μM, 24 h). The data in (**E**) are representative of three independent experiments. All the data are presented as mean ± SD. *p < 0.05, **p < 0.01, ns represents no significant difference.

The massive accumulation of dsDNA in the cytoplasm of cells triggers the activation of cytoplasmic DNA sensors, initiating a cascade of downstream innate immune pathways 31. Our analysis of RNA-seq data revealed significant activation of cytoplasmic DNA sensor pathways and pattern recognition receptor pathways in LV-D HCC cells (Figure 5A, B). Furthermore, GO analysis demonstrated activation of inflammatory and interferon (IFN) pathways in LV-D HCC cells (Figure 5C), suggesting that DNASE1L3 may play a role in activating cytoplasmic DNA sensors. Notably, cytoplasmic dsDNA receptors, such as cyclic GMP-AMP synthase (cGAS) and absent in melanoma 2 (AIM2), can localize to the nucleus to detect damaged DNA 32, 33. To validate whether DNASE1L3 enhances the activation of these cytoplasmic receptors, we examined the protein expressions associated with the stimulator of interferon response cGAMP interactor 1 (STING1) pathway and the AIM2 pathway. The expression levels of proteins related to the cGAS-STING1 pathway and AIM2 pathway were found to be higher in LV-D HCC cells compared to LV-veh HCC cells following sorafenib treatment (Figure 5D, E). Interferon regulatory factor 3 (IRF3), a downstream molecule of the cGAS-STING1 pathway, translocates to the nucleus upon phosphorylation by phosphorylated TANK-binding kinase 1 (p-TBK1) 34. We observed significant nuclear translocation of IRF3 in LV-D HCC cells after sorafenib stimulation (Figure 5F). Additionally, AIM2 oligomerizes upon recognition of cytoplasmic dsDNA and subsequently polymerizes with ASC to form an inflammasome 35. In LV-D HCC cells, we observed colocalization and polymerization of AIM2 with ASC, resulting in the formation of clusters (Figure 5G).

Recent studies have demonstrated that AIM2 serves as an initial protein in PANoptosis, forming the PANoptosome in conjunction with Pyrin, RIPK1, and other proteins 36 (Figure 5H). We observed the co-localization of Pyrin, RIPK1, and AIM2 in LV-D HCC cells, suggesting the formation of the PANoptosome (Figure 5I and S6A).

This finding was further corroborated by the Co-IP assay, which indicated the presence of these protein complexes (Figure 5J). Previous research has also reported that AIM2 can directly bind to dsDNA *via* its oligonucleotide-binding (OB) domain 37. Additionally, we observed the co-localization of γH2AX with the PANoptosome (Figure S6B). Furthermore, the Co-IP assay confirmed that AIM2 can bind to γH2AX and other PANoptotic proteins in sorafenib-treated LV-D HCC cells (Figure S6C).

Given that DNASE1L3 also activates the cGAS-STING1 pathway, we investigated its potential influence on the PANoptosis pathway. We performed cellular IF staining and observed that cGAS did not co-localize with the PANoptosome (Figure S6D), a finding further supported by the Co-IP assay (Figure S6E). Previous studies have indicated that the cGAS-STING1 pathway enhances the expression of major histocompatibility complex class I (MHC I) molecules 38. Our bioinformatics analysis revealed that patients with high *DNASE1L3* expression exhibited a significantly elevated antigen-presenting pathway involving MHC I molecules compared to those with low *DNASE1L3* expression (Figure S6F). Furthermore, we detected increased MHC I expression in LV-D HCC cells compare to LV-veh HCC cells (Figure S6G, H). These results suggest that DNASE1L3 may promote PANoptosis by activating the AIM2 pathway through enhanced dsDNA production. Additionally, it may also stimulate the cGAS-STING1 pathway to augment the antigen-presenting function *via* MHC I molecules.

### Knockdown of AIM2 reduces PANoptosis induced by DNASE1L3 in HCC cells

**Figure 6:**
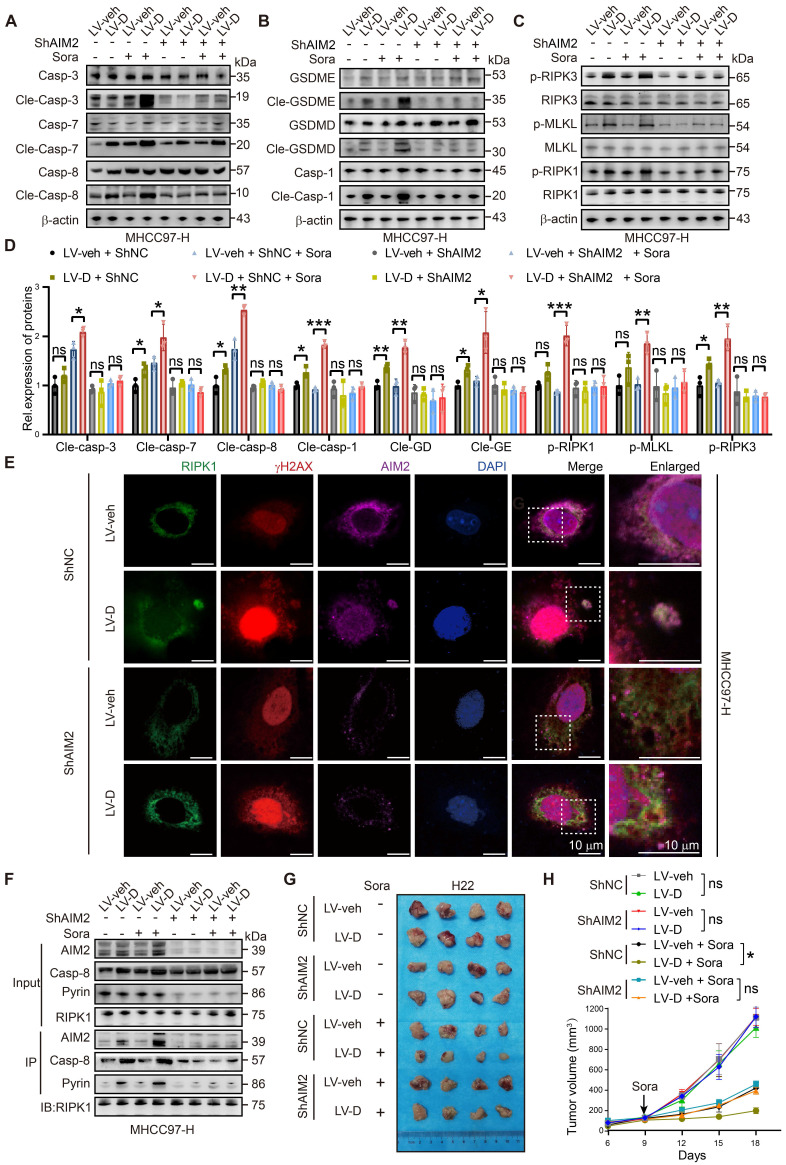
** Knockdown of AIM2 attenuates DNASE1L3-induced PANoptosis.** (**A-D**) Western blotting analysis of apoptosis-related proteins (**A**), pyroptosis-related proteins (**B**), necroptosis-related proteins (**C**) in LV-veh and LV-D MHCC97-H HCC cells with sorafenib (20 μM, 24 h), and the quantification of western blotting (**D**). (**E**) IF assay of RIPK1 (green), γH2AX (red), AIM2 (purple), and DAPI (blue) in ShNC, ShAIM2, LV-veh, and LV-D MHCC97-H HCC cells treated with sorafenib (20 μM, 24 h). The co-expression assembled spot represents PANoptosome. Scale bar = 10 μm. (**F**) Co-IP assay of Pyrin, RIPK1, AIM2, and caspase-8 in ShNC, ShAIM2, LV-veh, and LV-D MHCC97-H HCC cells with or without sorafenib (20 μM, 24 h). (**G**) Photographs of the excised tumors from nude mice injected with ShNC, ShAim2, LV-veh, and LV-D H22 mouse liver cancer cells subcutaneously. (**H**) Statistical analysis of the tumor volumes (mm^3^) of the H22 cells mouse model. Sorafenib treatment was applied beginning at the time pointed by the arrow. The data in (**D**) are representative of three independent experiments. The data in (**H**) are representative of four independent experiments. All the data are presented as mean ± SD. *p < 0.05, **p < 0.01, ***p < 0.001, ns represents no significant difference.

To validate our findings, we conducted experiments to knock down AIM2 and STING1 in LV-D and LV-veh HCC cells (Figure S7A, B). Our results indicated that the expression of PANoptosis-related proteins was elevated in sorafenib-treated LV-D cells. However, following the knockdown of AIM2, this expression was diminished and did not significantly differ from that observed in LV-veh cells (Figure 6A-D). Furthermore, our IF experiment revealed no evident formation of PANoptosomes in LV-D cells post-AIM2 knockdown (Figure 6E and S7C). This finding was corroborated by our Co-IP experiment, which demonstrated that the knockdown of AIM2 diminished the formation of the PANoptosis complex (Figure 6F). Conversely, the knockdown of STING1 in LV-D HCC cells resulted in a decrease in the expression of MHC I, without impacting the expression of PANoptosis-related proteins (Figure S7D-G). This suggests that the cGAS-STING1 pathway may not significantly contribute to DNASE1L3-promoted PANoptosis. Additionally, we established a subcutaneous tumor model in nude mice following the knockdown of AIM2 in LV-D and LV-veh mouse liver cancer cells. Our observations revealed that the knockdown of AIM2 decreased the sensitivity of LV-D mouse liver cancer cells to sorafenib, while exerting no significant effect on LV-veh cells (Figure 6G, H). Overall, our results suggest that DNASE1L3 enhances PANoptosis in HCC cells *via* the AIM2 pathway, and that knockdown of AIM2 attenuates PANoptosis induced by DNASE1L3.

### DNASE1L3 promotes inflammatory pathways and immune cells infiltration in TME

**Figure 7:**
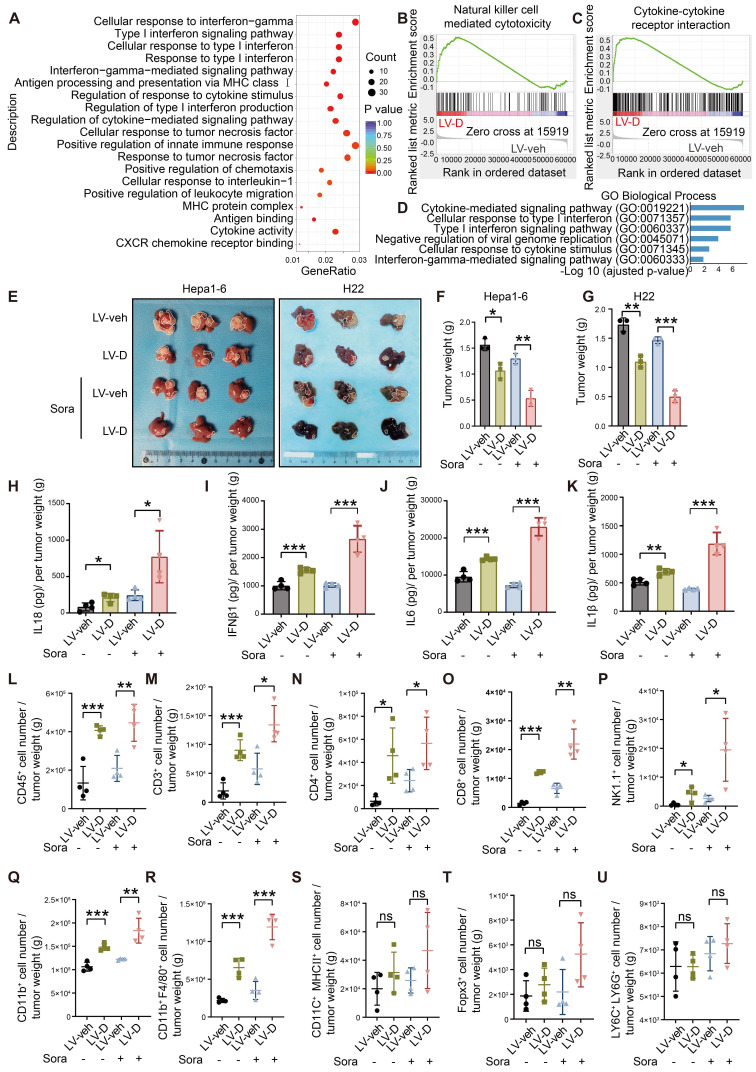
** DNASE1L3-induced PANoptosis promotes anti-tumor immunity in TME.** (**A**) GO enrichment analysis of DEGs between LV-veh and LV-D MHCC97-H HCC cells. (**B** and **C**) Representative GSEA in RNA-seq from LV-veh and LV-D MHCC97-H HCC cells. (**D**) GO enrichment analysis of DEGs from liver cancer tissues with high or low expression of *DNASE1L3* in the TCGA database. (**E**) Photographs of the excised livers from LV-veh and LV-D Hepa1-6 and H22 liver cancer cells constructed liver orthotopic tumor implantation mouse model. (**F** and **G**) Statistical analysis of the tumor weight (g) of the Hepa1-6 model (**F**) and H22 model (**G**). (**H**-**K**) The mouse inflammatory factors in liver orthotopic tumor with sorafenib treatment including IL18, IFNβ1, IL6, and IL1β were detected by ELISA. Statistical analysis was calculated per tumor weight. (**L**-**U**) Immune phenotyping was performed using FCM using the indicated cell markers in LV-veh and LV-D Hepa1-6 liver cancer cells constructed liver orthotopic tumors, collected at day 35. The data in (**F**, **G**) are representative of three independent experiments. The data in (**H**-**U**) are representative of four independent experiments. All the data are presented as mean ± SD. *p < 0.05, **p < 0.01, ***p < 0.001, ns represents no significant difference.

PANoptosis, a form of inflammatory PCD, initiates the release of cellular contents and inflammatory factors, thereby activating the inflammatory response within the TME 9. Our RNA-seq analysis demonstrated a significant upregulation of inflammatory factors and innate immune pathways in LV-D HCC cells (Figure 7A-C). Additionally, bioinformatics analysis revealed that patients exhibiting high expression levels of *DNASE1L3* had significantly elevated inflammatory factor pathways compared to those with low* DNASE1L3* expression (Figure 7D). To investigate the role of DNASE1L3 in the TME, we performed liver orthotopic tumor implantation experiments using immune-competent C57BL/6 or BALB/c mice. Notably, in contrast to the previous subcutaneous tumor model using nude mice, the growth rate of LV-D orthotopic tumors was significantly lower than that of LV-veh orthotopic tumors, with this difference becoming even more pronounced following sorafenib treatment (Figure 7E-G). These findings suggest that DNASE1L3 may influence the proliferation of liver cancer cells through immune mechanisms within the TME. To further elucidate this, we evaluated the release of inflammatory factors in LV-D and LV-veh HCC cells. Specifically, we quantified the levels of four inflammatory factors—Interleukin 18 (IL18), IL1β, IFNβ1, and IL6—associated with the cGAS-STING1 and AIM2 pathways. Our results indicated a significant increase in these inflammatory factors in LV-D HCC cells compared to LV-veh HCC cells, with this difference being even more pronounced after sorafenib treatment (Figure S8A-E). Consistent results were also observed in the levels of these inflammatory factors in the orthotopic tumors of mice (Figure 7H-K).

Based on the aforementioned results, we investigated the impact of DNASE1L3 expression in liver cancer cells on immune cell infiltration within the TME. Our study utilized mice with orthotopic tumors, and we observed a variety of inflammatory cell types. Notably, our findings indicated that the populations of CD4^+^ T cells, CD8^+^ T cells, natural killer (NK) cells, and macrophages were significantly elevated in LV-D orthotopic tumors compared to LV-veh orthotopic tumors (Figure 7L-U and S8F). These results suggest that DNASE1L3 enhances the release of pro-inflammatory factors and facilitates the infiltration of anti-tumor immune cells following sorafenib treatment. As a consequence, this promotes the establishment of a more conducive anti-tumor TME and contributes to the inhibition of tumor growth.

### DNASE1L3 enhances the efficacy of sorafenib combined with PD-1 monotherapy which is attenuated by knockdown of AIM2

**Figure 8:**
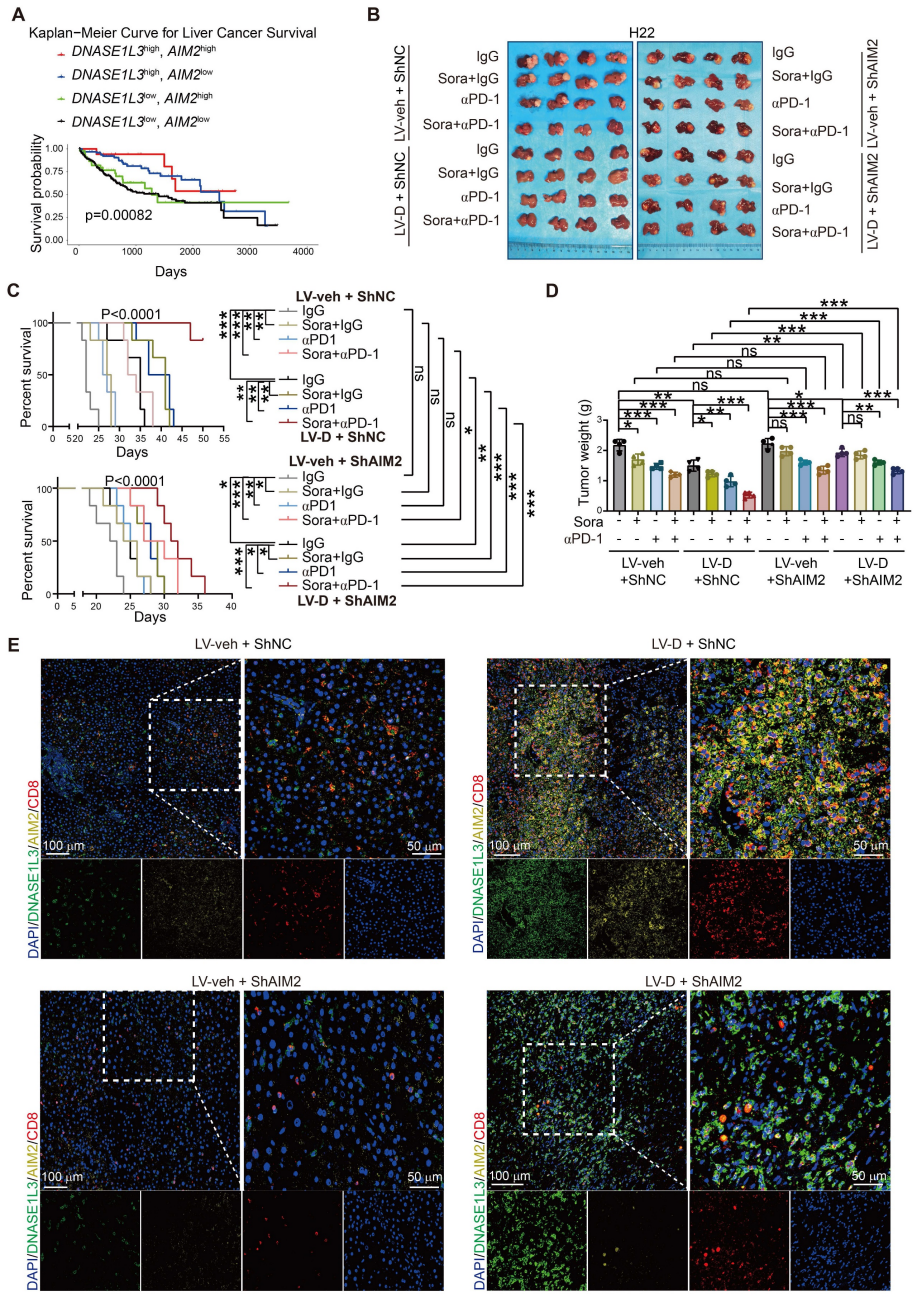
** DNASE1L3 improves the effectiveness of combination therapy with sorafenib and PD-1 mAb.** (**A**) Kaplan-Meier survival curve based on the relative expression of *DNASE1L3* and *AIM2* in liver cancer tissues from the TCGA database. (**B**) Photographs of the excised livers from H22 liver cancer cells constructed liver orthotopic tumor implantation mouse model treated by sorafenib monotherapy, αPD-1 monotherapy and combination therapy with sorafenib and αPD-1. (**C**) Survival curve of H22 liver cancer cells constructed liver orthotopic tumor implantation mouse model treated by sorafenib monotherapy, αPD-1 monotherapy and combination therapy with sorafenib and αPD-1. (**D**) Statistical analysis of the tumor weight (g) of H22 liver cancer cells constructed liver orthotopic tumor implantation mouse model treated by sorafenib monotherapy, αPD-1 monotherapy and combination therapy with sorafenib and αPD-1. (**E**) IF assay was used to verify the expression of DAPI (blue), DNASE1L3 (green), AIM2 (yellow) and CD8 (red) for the H22 liver cancer cells constructed liver orthotopic tumor implantation mouse model treated by combination therapy with sorafenib and αPD-1. Scale bars in overall images (left) is 100 μm, and in enlarged images (right) is 50 μm. Images with separated channels are below. The data in (**C**) are representative of six independent experiments. The data in (**D**) are representative of four independent experiments. All the data are presented as mean ± SD. *p < 0.05, **p < 0.01, ***p < 0.001, ns represents no significant difference.

We also investigated the relationship between DNASE1L3 and AIM2 concerning the survival of HCC patients. Bioinformatics analysis revealed that patients exhibiting high expressions of both *DNASE1L3* and *AIM2* genes experienced the longest survival times, whereas those with low expressions of both genes had the shortest survival (Figure 8A). This finding suggests that the concurrent high expression of *DNASE1L3* and *AIM2* may be linked to improved prognosis. Conversely, patients with high expressions of both *DNASE1L3* and *STING1* did not display a significant survival advantage when compared to patients with elevated *DNASE1L3* expression and low *STING1* expression (Figure S9A).

In addition, we established a liver orthotopic tumor implantation mouse model to investigate the effects of sorafenib monotherapy, PD-1 mAb therapy, and the combination of both treatments. After confirming the formation of orthotopic tumors through *in vivo* imaging, we administered these therapies to the mice and monitored changes in tumor size and survival (Figure S9B). Our results indicated that orthotopic tumors overexpressing DNASE1L3 exhibited greater responsiveness to the combination of sorafenib and PD-1 mAb, as evidenced by reduced tumor weight and extended survival compared to the monotherapy group (Figure S9C-E). Conversely, the knockdown of AIM2 in the LV-D group resulted in increased tumor weight and decreased survival, suggesting that DNASE1L3 enhances the efficacy of the combination therapy *via* the AIM2 pathway. We also assessed the impact of PD-1 monotherapy; notably, the overexpression of DNASE1L3 significantly improved the efficacy of both sorafenib monotherapy and αPD-1 monotherapy, with the most pronounced effect observed in the combination therapy (Figure 8B-D). However, after αPD-1 monotherapy, we did not observe a significant increase in anti-tumor immune factors within the tumor tissue when compared to the untreated group. This finding implies that αPD-1 monotherapy does not enhance efficacy through the direct promotion of anti-tumor immune factor release in the microenvironment, contrasting with the mechanism of action noted with sorafenib (Figure S9F-I). Furthermore, IF staining of tumor tissues in the combination therapy group revealed that CD8^+^ T cell infiltration was higher in tissues with elevated expression of DNASE1L3 and AIM2, whereas the knockdown of AIM2 led to a significant reduction in CD8^+^ T cell numbers. Tumors exhibiting low expression of DNASE1L3 demonstrated minimal CD8^+^ T cell infiltration (Figure 8E).

On the side, we assessed CD8^+^ T cell infiltration across the untreated, sorafenib monotherapy, αPD-1 monotherapy, and combination therapy groups. Our analysis revealed that CD8^+^ T cell infiltration was more pronounced in the groups exhibiting high expression levels of DNASE1L3 and AIM2, with the most significant difference observed in the combination therapy group (Figure S9J-M and S10A, B). This finding suggests that DNASE1L3 plays critical a role in enhancing the immune TME, particularly within the context of combination therapy for HCC. Based on these results, we conclude that elevated expression of DNASE1L3 and AIM2 improve the efficacy of sorafenib in conjunction with PD-1 mAb therapy, resulting in significant tumor growth inhibition and extended survival in mice.

## Discussion

The role of deoxyribonucleases in tumor immunity and therapy is of significant interest; however, it remains largely underexplored. Notably, high expression levels of DNASE1L3 are positively correlated with improved prognosis in HCC, yet its impact on treatment outcomes in HCC has not been thoroughly investigated. Yang *et al.* demonstrated that DNASE1L3 is associated with longer survival following radical resection of HCC 21. Furthermore, Guo *et al.* reported that the inhibition of DNASE1L3 in HCC cells increased resistance to sorafenib, although the underlying mechanisms were not explored 23. In this study, we identify DNASE1L3 as a biomarker indicative of better prognosis for patients undergoing sorafenib combined with PD-1 mAb therapy in HCC, and elucidate the mechanisms involved.

Our findings suggest that DNASE1L3 functions as a deoxyribonuclease, activating the AIM2 pathway through the generation of dsDNA which in turn triggers inflammatory PCD known as PANoptosis. Additionally, our analysis of liver cancer datasets indicates that DNASE1L3 is positively correlated with improved prognosis for both sorafenib and αPD-1 monotherapy. Currently, the available liver cancer datasets do not provide information on the prognosis of combination therapies; thus, we plan to establish a cohort to address this gap in the future. Overall, our study offers insights into potential biomarker molecules for predicting outcomes of combination therapy in advanced HCC and clarifies the mechanism by which DNASE1L3 promotes PANoptosis in HCC cells.

Targeted therapy combined with immunotherapy represents a promising strategy for tumor treatment. However, many patients do not respond to this therapeutic approach, and the underlying reasons for this lack of response have not been thoroughly investigated 39. Our study performed a proteomic analysis on HCC tissues from patients who either responded or did not respond to sorafenib in conjunction with PD-1 mAb therapy. We identified that DNASE1L3, a specific protein, was significantly upregulated. Previous research has demonstrated that other DNA enzymes, such as three prime repair exonuclease 1 (TREX1) and Deoxyribonuclease 2 (DNASE2), facilitate dsDNA degradation and inhibit cGAS-STING1 pathway activation 40, 41. However, our experiments indicated that HCC cells overexpressing DNASE1L3 exhibit accelerated DNA cleavage and accumulate higher levels of dsDNA in the cytoplasm following sorafenib treatment. We hypothesize that this discrepancy may be attributed to the distinct types of DNASE involved and the diverse products resulting from cleavage. Unlike other DNASEs, DNASE1L3 performs different functions both intracellularly and extracellularly. It primarily digests macromolecular chromosome fragments, including apoptotic vesicles and exosomes, in the extracellular space to mitigate the formation of autoantibodies. Mutational inactivation of DNASE1L3 is a prevalent cause of autoimmune diseases, such as lupus erythematosus 42. Conversely, DNASE1L3 predominantly functions intracellularly to cleave chromatin DNA into nucleosome-length (166 bp) dsDNA fragments during cell death 43, 44. In our study, we concentrated on a detailed investigation of the intracellular function of DNASE1L3; however, we did not explore its extracellular effects within the TME in this research. These extracellular effects will be addressed in future studies.

DNA is typically localized within the nucleus as chromatin. The release of damage-associated molecular patterns (DAMPs) during early cell death can significantly influence the type of cell death 45, 46. Our study found that DNASE1L3 induces the production of dsDNA, which activates the cytoplasmic sensors AIM2 and cGAS, initiating PANoptosis. Cytoplasmic dsDNA sensors are conserved mechanisms that detect endogenous nucleic acids during microbial infection or cell death, thereby initiating innate immune signaling pathways 47, 48. Common sensors include AIM2 35, cGAS 49, Toll-like receptor 9 (TLR9) 50, and others. TLR9 is primarily located in immune cells such as dendritic cells (DCs), B cells, and macrophages, where it recognizes the prevalence of non-methylated cytosine-guanine (CpG) motifs in bacterial and viral DNA 51. In contrast, AIM2 and cGAS bind directly to dsDNA in a sequence-independent manner, with AIM2 recognizing dsDNA lengths greater than 80 base pairs (bp) and cGAS recognizing lengths above 45 bp 32, 35. Our experiments demonstrated that both AIM2 and cGAS-STING1 pathways were activated; however, knockdown of AIM2 resulted in decreased PANoptosis, while knockdown of STING1 did not significantly affect PANoptosis. Previous studies have explored the crosstalk between AIM2 and STING1 pathways, indicating that the cGAS-STING1 pathway can enhance the expression of AIM2 and caspase-1 through IFNγ 52. Conversely, other studies suggest a suppressive interaction between the cGAS-STING1 and AIM2 pathways 53. Nevertheless, further validation is required to confirm these findings.

One reason for the low efficacy of immunotherapy in HCC is its reduced immunogenicity, often referred to as a 'cold tumor' 54. A critical challenge in enhancing the effectiveness of immunotherapy lies in identifying methods to increase the immunogenicity of HCC and convert it into a 'hot tumor' 55. Transitioning tumor cell death from low immunogenic apoptosis to high immunogenic cell death appears promising in this context 56. Previous research has predominantly concentrated on the effects of sorafenib on apoptosis in HCC cells 25. However, in HCC cells that overexpressing DANSE1L3, sorafenib induces PANoptosis rather than apoptosis. PANoptosis, an inflammatory form of PCD, results in the release of pro-inflammatory factors. Furthermore, dsDNA activates the cGAS-STING1 pathway, which promotes the expression of downstream MHC I molecules and enhances the immunogenicity of HCC cells. In a liver orthotopic tumor implantation mouse model, we observed that LV-D tumors exhibited a higher presence of anti-tumor immune cells and demonstrated improved prognosis when treated with a combination of sorafenib and PD-1 mAb. Nevertheless, the role of PANoptosis within the TME has not been extensively investigated. In future studies, we aim to further examine the characteristics of PANoptosis in the TME and establish a link between PANoptosis and anti-tumor immunity.

Our findings present a novel strategy to enhance the effectiveness of anti-HCC therapy. However, it is important to acknowledge several limitations of this study. Firstly, sorafenib, as a TKI, targets multiple pathways within tumors 57. While we observed a correlation between the expression of DNASE1L3 and PCD in HCC cells, additional research is necessary to investigate the angiogenesis-related pathways within the TME. Secondly, DNASE1L3 is known to be secreted extracellularly, and existing studies suggest its role in the formation of extracellular traps 58. Thus, the extracellular function of DNASE1L3 in the TME warrants further exploration. Thirdly, the relationship between PANoptosis and metabolic rate remains ambiguous. Previous literature has indicated that DNASE1L3 inhibits glycolysis 18, and our experiments also revealed that the overexpression of DNASE1L3 significantly disrupts the oxidative respiratory chain in response to sorafenib induction. However, the role of PANoptosis in the metabolism of HCC cells requires further investigation. Lastly, we encountered limitations in sample selection due to the scarcity of biopsy samples from patients undergoing systemic treatment and those receiving adjuvant therapy for HCC. Consequently, we focused on cases of primary tumors and early recurrences for this study, which may raise concerns regarding tumor heterogeneity. Some studies suggest that early recurrence is more likely a consequence of micrometastasis from the primary tumor 59, and that organoids derived from primary tissue can be utilized to screen for sensitive therapeutic agents following recurrence 60. Moving forward, we will continue to collect data from patients undergoing adjuvant therapy and aim to expand our sample size to further validate these findings.

In conclusion, our study has identified DNASE1L3 as a potential biomarker for predicting the sensitivity of combination therapy that involves sorafenib and immunotherapy in HCC. Additionally, we have explored the underlying mechanism by which DNASE1L3 enhances the efficacy of this combination therapy through the activation of AIM2-mediated PANoptosis. Collectively, our findings indicate that heightening DNASE1L3 may represent a promising therapeutic strategy for improving the antitumor efficacy of combination therapy in the treatment of HCC.

## Materials and methods

### Human surgical specimens

This study utilized a variety of human surgical specimens, including eight primary HCC tumors from patients who underwent combination therapy with sorafenib and PD-1 mAb, eight primary HCC tumors from patients treated exclusively with sorafenib, and twelve pairs of primary HCC tumors compared to adjacent normal tissues. Importantly, there was no overlap among the patients in the three cohorts. To identify effective biomarker molecules associated with both combination therapy and sorafenib monotherapy, we performed a retrospective analysis of patients who were hospitalized for radical surgery for HCC at the Second Affiliated Hospital of the Army Medical University between January 2019 and December 2021. The inclusion criteria were as follows: (1) Clinically confirmed HCC based on clinical criteria or histological examination; (2) Radical resection of primary HCC tumors with retained samples for testing; (3) Early recurrence, defined as occurring within two years, resulting in unresectable tumors; (4) Presence of at least one measurable tumor lesion according to the RECIST 1.1 criteria; (5) Patients received regular treatment for a minimum of six months, with accessible clinical data from medical records; (6) No history of other malignant cancers. Based on these criteria, we identified eight patients receiving combination therapy and eight patients receiving sorafenib monotherapy, categorizing them into responding and poor-responding groups according to the RECIST 1.1 criteria. The use of clinical specimens in this study was approved by the Ethical Review Board of the Second Affiliated Hospital ethics committee.

### Cell culture and treatment

Human liver cell lines L-02 (Cat#BFN608006124), WRL68 (Cat#BFN608007148), HCC cell lines BEL-7402 (Cat#BFN60800694), SMMC-7721 (Cat#BFN60800687), and mouse liver cancer cell lines H22 (Cat#BFN608007276) were purchased from BluefBio (China). Human HCC cell lines HepG2 (Cat#SCSP-510), HuH-7 (Cat#SCSP-526), MHCC97-H (Cat#SCSP-5092), HCCLM3 (Cat#SCSP-5093), PLC/PRF/5 (Cat#TCHu119), Hep3B (Cat#SCSP-5045), and mouse liver cancer cell lines Hepa1-6 (Cat#SCSP-512) were obtained from the Cell Bank of Chinese Academy of Sciences (CAS). All cell lines used in this study were authenticated and tested for mycoplasma contamination. Cell passages with a value of less than ten were utilized. Except for H22, all cell lines were cultured in DMEM (Cat#11965092, Gibco, USA) supplemented with 10% fetal bovine serum (Cat#A3160902, Gibco, USA) and penicillin/streptomycin (10000 U/ml, Cat#15070063, Thermo scientific, USA) in a 5% CO_2_ atmosphere at 37°C.

### Animal experiments

All animal experimental protocols were approved by the Army Medical University Animal Ethics Committee, under Animal Ethics Number AMUWEC20224407. The protocols strictly adhered to the National Institutes of Health guidelines concerning animal welfare. For the subcutaneous tumor model in nude mice (Cat#401, Vital River, China), H22 and Hepa1-6 mouse liver cancer cells (5×10^5^ cells of H22 and 1×10^6^ cells of Hepa1-6) were injected subcutaneously into the lower right flank of male BALB/c nude mice aged 6-8 weeks. In the H22 subcutaneous tumor model, sorafenib treatment (5 mg/kg) (Cat#HY-10201, MedChemExpress, USA) was administered intraperitoneally on days 9, 12, and 15 post-injection. In the Hepa1-6 subcutaneous tumor model, sorafenib was administered intraperitoneally on days 12, 15, and 18 post-injection. Tumor diameter and width were measured every three days, and tumor volumes were calculated using the formula V = π/6 × L × W × H (where V is volume, L is length, W is width, and H is height). For the liver orthotopic tumor implantation mouse model, H22 and Hepa1-6 subcutaneous tumors were cut into small pieces (1 mm³) and implanted separately into the livers of BALB/c (Vital River, China) and C57BL/6 (Vital River, China) mice after anesthesia with pentobarbital sodium (Cat#57-33-0). Following six days of H22 orthotopic tumor implantation and twenty-five days of Hepa1-6 orthotopic tumor implantation, *in vivo* imaging analysis was conducted to assess the volume of the orthotopic tumors. Subsequently, sorafenib (5 mg/kg), PD-1 (Cat#BE0273, BioXcell) (200 µg per mouse), and a combination therapy of sorafenib and PD-1 antibody were administered intraperitoneally three times (every three days) prior to sacrifice.

### Proteomics analyses

Proteins were extracted from surgical specimens of HCC and subsequently subjected to label-free quantitative proteomics analysis (Jingjie PTM BioLab Co. Inc., China). Following this, a comprehensive bioinformatics analysis was conducted on all identified proteins, which included quantification of protein expression and differential expression analysis. Furthermore, the functions of the proteins were classified through GO and KEGG enrichment analyses, focusing on the differentially expressed proteins (DEPs).

### Statistical analysis

Statistical analyses were conducted using Prism software (version 9.00, GraphPad). Data are presented as mean ± SEM. Normality and lognormality were assessed using the Shapiro-Wilk test. An unpaired t-test was employed to compare the means of two groups exhibiting normal (or approximately normal) distributions. For comparisons involving more than two groups at different time points, two-way *ANOVA* with appropriate multiple comparison corrections (Dunnett's or Tukey's test) was utilized. Survival curves were analyzed using the Kaplan-Meier method, complemented by the log-rank test. Nonlinear fit analysis was applied in inhibition experiments, and IC_50_ values were derived from Best-fit estimates. All statistical tests were two-tailed, with statistical significance set at p values < 0.05.

## Supplementary Material

Supplementary materials and methods, figures and tables.

## Abbreviations

AIM2: absent in melanoma 2
ASC: apoptosis-associated speck-like protein
ATP: adenosine triphosphate
cGAS: cyclic GMP-AMP synthase
Co-IP: co-immunoprecipitation
CpG: non-methylated cytosine-guanine
DAMPs: damage-associated molecular patterns
DCs: dendritic cells
DEGs: differentially expressed genes
DNASE1L3: deoxyribonuclease 1 like 3
DNASE2: deoxyribonuclease 2
dsDNA: double-strand DNA
FCCP: carbonyl cyanide 4-(trifluoromethoxy)phenylhydrazone
GO: gene ontology
IC50: half-maximal inhibitory concentration
IF: immunofluorescence
IL18: interleukin 18
IRF3: interferon regulatory factor 3
LIHC: liver hepatocellular carcinoma
mAb: monoclonal antibody
MHC I: major histocompatibility complex class I
NK: natural killer
OB: oligonucleotide-binding
OCR: oxygen consumption rate
PCD: programmed cell death
PD-1: programmed death-1
p-TBK1: phosphorylated TANK-binding kinase 1
RECIST: response Evaluation Criteria in Solid Tumors Version
RIPK1: receptor-interacting protein kinase 1
ROS: reactive oxygen species
STING1: stimulator of interferon response cGAMP interactor 1
TCGA: the Cancer Genome Atlas
TKI: tyrosine kinase inhibitor
TLR9: toll-like receptor 9
TME: tumor microenvironment
TREX1: three prime repair exonuclease 1
VEGFR: vascular endothelial growth factor receptor
γH2AX: phosphorylated H2AX histone protein
DEPs: differentially expressed proteins
