# Supplementary material for: DNASE1L3-mediated PANoptosis enhances the efficacy of combination therapy for advanced hepatocellular carcinoma
Source: Theranostics. 2024 Oct 14;14(17):6798–817. doi: 10.7150/thno.102995 (PMC11519790; doi:10.7150/thno.102995)
Supplement: Supplementary file 1 — Supplementary materials and methods, figures and tables. [file thnov14p6798s1.pdf]

Supplementary Materials for

DNASE1L3-mediated PANoptosis enhances the efficacy of combination therapy for advanced hepatocellular carcinoma

Jingchun Wang[1], Yu Chen[2], Yanquan Xu[3], Jiangang Zhang[2], Shuai Yang[4], Yu Zhou[2], Juan Lei[2], Ran Ren[2], Yang Chen[2], Huakan Zhao[2]*, Yongsheng Li[2]*, Shiming Yang[1]*

***Corresponding author emails**: (lys@cqu.edu.cn) (Y.L.), (hk_zhao@cqu.edu.cn) (H.Z.), and (yangshiming@tmmu.edu.cn) (S.Y.)

This PDF file includes:

Figures S1 to S10

Tables S1 to S3

Materials and Methods

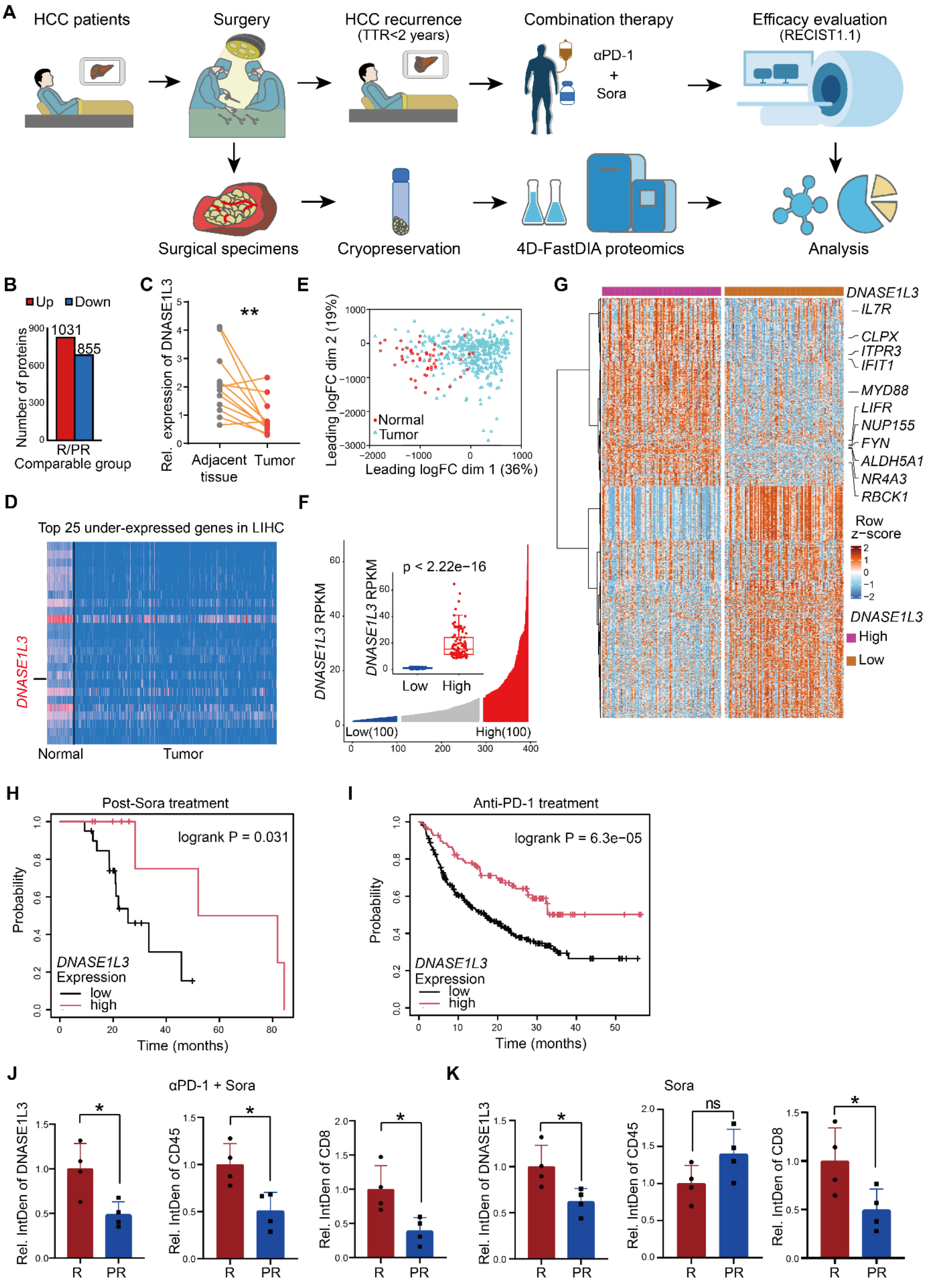
A
HCC patients
Surgery
HCC recurrence
(TTR<2 years)
Combination therapy
αPD-1
+
Sora
Efficacy evaluation
(RECIST1.1)
Surgical specimens
Cryopreservation
4D-FastDIA proteomics
Analysis
B
Up
Down
1031
855
Number of proteins
R/PR
Comparable group
C
Rel. expression of DNASE1L3
**
Adjacent tissue
Tumor
E
Leading logFC dim 2 (19%)
Leading logFC dim 1 (36%)
Normal
Tumor
G
DNASE1L3
IL7R
CLPX
ITPR3
IFIT1
MYD88
LIFR
NUP155
FYN
ALDH5A1
NR4A3
RBCK1
Row z−score
DNASE1L3
High
Low
D
Top 25 under-expressed genes in LIHC
DNASE1L3
Normal
Tumor
F
DNASE1L3 RPKM
p < 2.22e−16
Low
High
Low(100)
High(100)
H
Post-Sora treatment
logrank P = 0.031
Probability
DNASE1L3
Expression
low
high
Time (months)
I
Anti-PD-1 treatment
logrank P = 6.3e−05
Probability
DNASE1L3
Expression
low
high
Time (months)
J
αPD-1 + Sora
Rel. IntDen of DNASE1L3
Rel. IntDen of CD45
Rel. IntDen of CD8
*
R
PR
K
Sora
Rel. IntDen of DNASE1L3
Rel. IntDen of CD45
Rel. IntDen of CD8
ns
*
R
PR

Supplementary Figure S1. DNASE1L3 is downregulated in HCC tissue. (**A**) Flow diagram of surgical specimens from HCC patients detected by proteomics analysis. (**B**) Number of proteins differentially expressed in responsive (R) and poor-responsive (PR) patients detected by proteomics analysis. (**C**) The protein expression of DNASE1L3 in HCC tumor tissues versus adjacent non-cancerous tissues. (**D**) The top 25 under-expressed genes in liver hepatocellular carcinoma (LIHC) (from the TCGA database). *DNASE1L3* was represented in red color. (**E**) The principal component analysis (PCA) scatterplot is the scatterplot of gene expression correlation between samples. The horizontal and vertical coordinates represent log10[RPKM] of different sample genes, respectively. (**F**) The large image illustrates the clustering of samples based on the expression levels of *DNASE1L3*, with the samples arranged sequentially according to their *DNASE1L3* expression. The vertical axis denotes the RPKM values for *DNASE1L3* expression. In the smaller figure, each point corresponds to an individual sample. The significance of the differences in *DNASE1L3* expression among the various groups is indicated above, utilizing the Wilcoxon test as the statistical method. (**G**) The hierarchical clustering tree of coordinates illustrates the overall hierarchical clustering of DEGs. In the primary visualization, the normalized Reads Per RPKM relative expression values of genes, represented as z-scores, are displayed. High-expressed genes are indicated in red, while low-expressed genes are shown in blue. The x-axis represents the various samples, whereas the y-axis lists the gene names. Immune-related genes are noted on the right side of the figure. (**H** and **I**) Kaplan-Meier survival curve based on the relative expression of DNASE1L3 and post-treatment of sorafenib (**H**) and anti-PD-1 immunotherapy (**I**) from the Kaplan-Meier Plotter website. (**J** and **K**) The IntDen level of DNASE1L3, CD45 and CD8 in IF of tumor tissues from patients responsive (R) or poor-responsive (PR) to combination therapy (**J**) and sorafenib monotherapy (**K**). The data in (**J**, **K**) are representative of four

independent experiments. All the data are presented as mean ± SD. $^*p < 0.05$, $^{**}p < 0.01$, ns represents no significant difference.

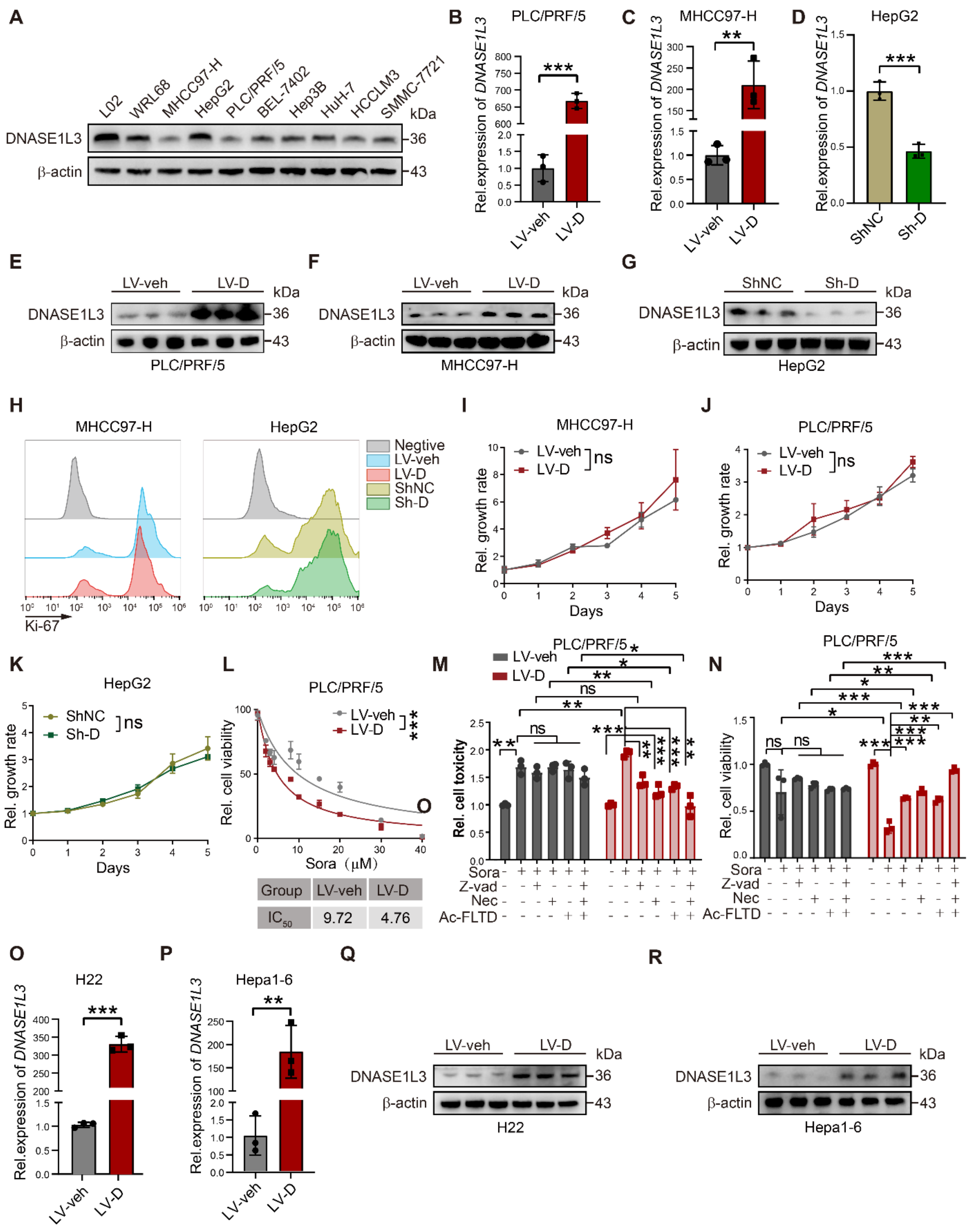

Group	LV-veh	LV-D
IC_{50}	9.72	4.76

Supplementary Figure S2. DNASE1L3 promotes sorafenib sensitivity in HCC cells. (A)

Western blotting analysis of DNASE1L3 in different human liver and HCC cell lines. (**B**-**D**) RT-qPCR presented the *DNASE1L3* expression after overexpression of *DNASE1L3* in MHCC97-H (**B**), PLC/PRF/5 HCC cells (**C**), and knockdown in HepG2 HCC cells (**D**). (**E**-**G**) Western blotting analysis of DNASE1L3 after overexpression in MHCC97-H (**E**), PLC/PRF/5 HCC cell lines (**F**) and knockdown in HepG2 HCC cells (**G**). (**H**) Flow cytometry (FCM) analysis for Ki-67 expression among the LV-veh, LV-D MHCC97-H HCC cells, and ShNC, Sh-D HepG2 HCC cells. (**I**-**K**) CCK8 proliferation analysis of LV-veh, LV-D MHCC97-H HCC cells (**I**), LV-veh and LV-D PLC/PRF/5 HCC cells (**J**), and ShNC, Sh-D HepG2 HCC cells (**K**). (**L**) CCK8 sensitivity analysis with sorafenib concentration gradient from 0 μM to 60 μM in LV-veh and LV-D PLC/PRF/5 HCC cells for 48 h. (**M** and **N**) Cell toxicity (**M**) and viability (**N**) assessments of LV-veh, LV-D PLC/PRF/5 HCC cells treated or not treated with 10 μM sorafenib for 48 h in combination with the apoptosis inhibitor Z-VAD-FMK (VAD, 25 μM), the necroptosis inhibitor necrostatin (Nec, 20 μM), and the pyroptosis inhibitors Ac-FLTD (20 μM). (**O** and **P**) RT-qPCR present the *DNASE1L3* expression after overexpression of *DNASE1L3* in H22 (**O**) and Hepa1-6 mouse liver cancer cells (**P**). (**Q** and **R**) Western blotting analysis of DNASE1L3 after overexpression in H22 (**Q**) and Hepa1-6 mouse liver cancer cells (**R**). The data in (**B**-**D**, **I**-**P**) are representative of three independent experiments and each sample has five replication wells. All the data are presented as mean ± SD. $^{*}p < 0.05$, $^{**}p < 0.01$, $^{***}p < 0.001$, ns represents no significant difference.

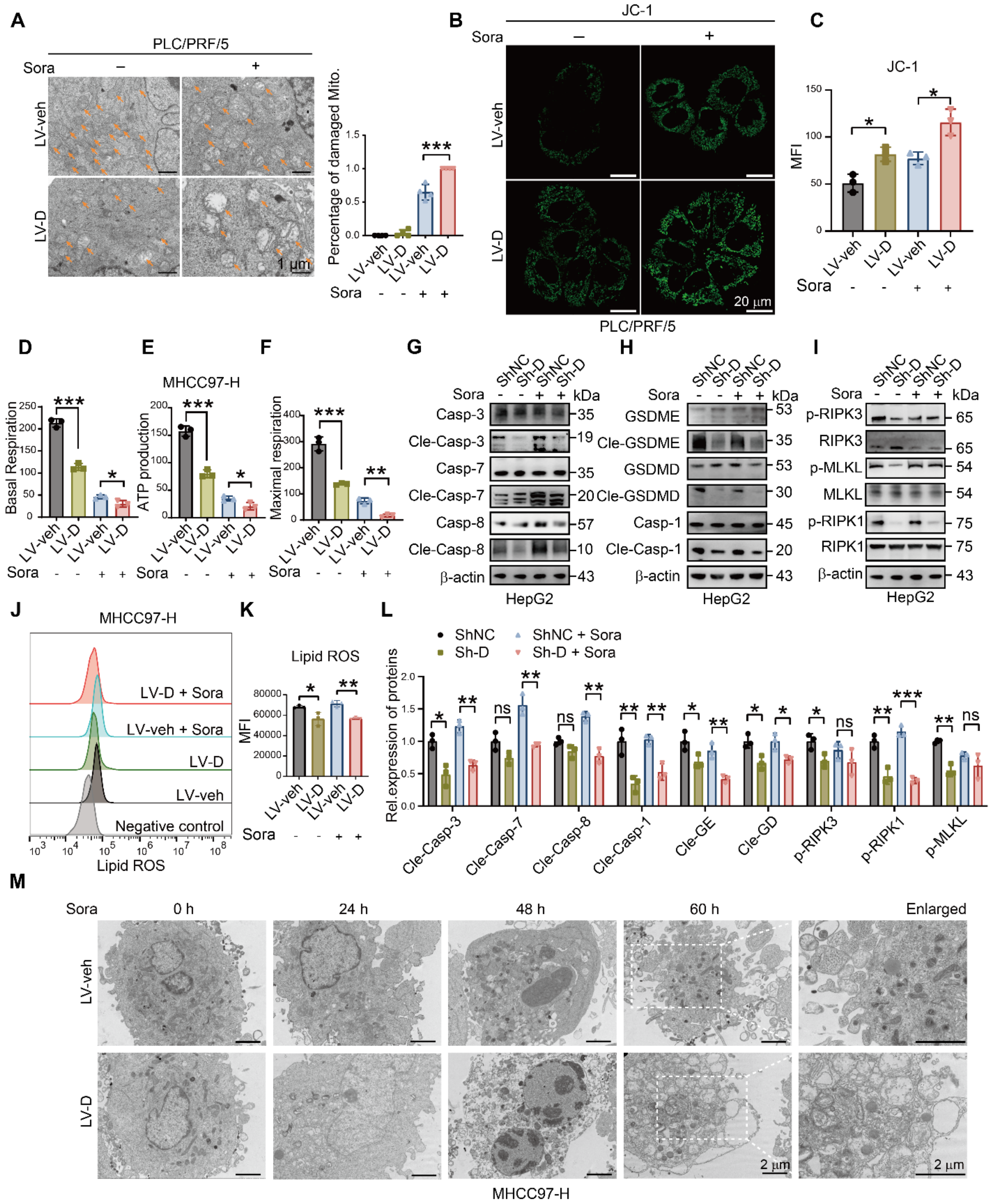

Supplementary Figure S3. **DNASE1L3 induces PANoptosis after sorafenib treatment.** (A) Electron microscope images of LV-veh and LV-D PLC/PRF/5 HCC cells treated with sorafenib

(10 μM, 24 h). Mitochondria were represented by orange arrows. Scale bar = 1 μm. (**B**) Mitochondria membrane potential was represented by the intensity of the green image. Scale bar = 20 μm. (**C**) Quantification of the intensity of mitochondria membrane potential. (**D**-**F**) Quantification of the basal respiration (**D**), ATP production (**E**) and maximal respiration (**F**) of seahorse assay in LV-veh and LV-D MHCC97-H HCC cells treated with or without sorafenib (20 μM, 24 h). (**G**-**I**) Western blotting analysis of apoptosis-related proteins (**G**), pyroptosis-related proteins (**H**), and necroptosis-related proteins (**I**) in ShNC and Sh-D HepG2 HCC cells treated with or without sorafenib (15 μM, 24 h). (**J** and **K**) Lipid ROS detected by FSM in LV-veh and LV-D MHCC97-H HCC cells treated with or without sorafenib (20 μM, 24 h). (**L**) Quantification of the western blotting analysis. (**M**) Time-dependent electron microscope images of LV-veh and LV-D MHCC97-H HCC cells treated with sorafenib (20 μM). Scale bar = 2 μm. The data in (**C**-**F**, **K**, **L**) are representative of three independent experiments. All the data are presented as mean ± SD. *$p < 0.05$, **$p < 0.01$, ***$p < 0.001$, ns represents no significant difference.

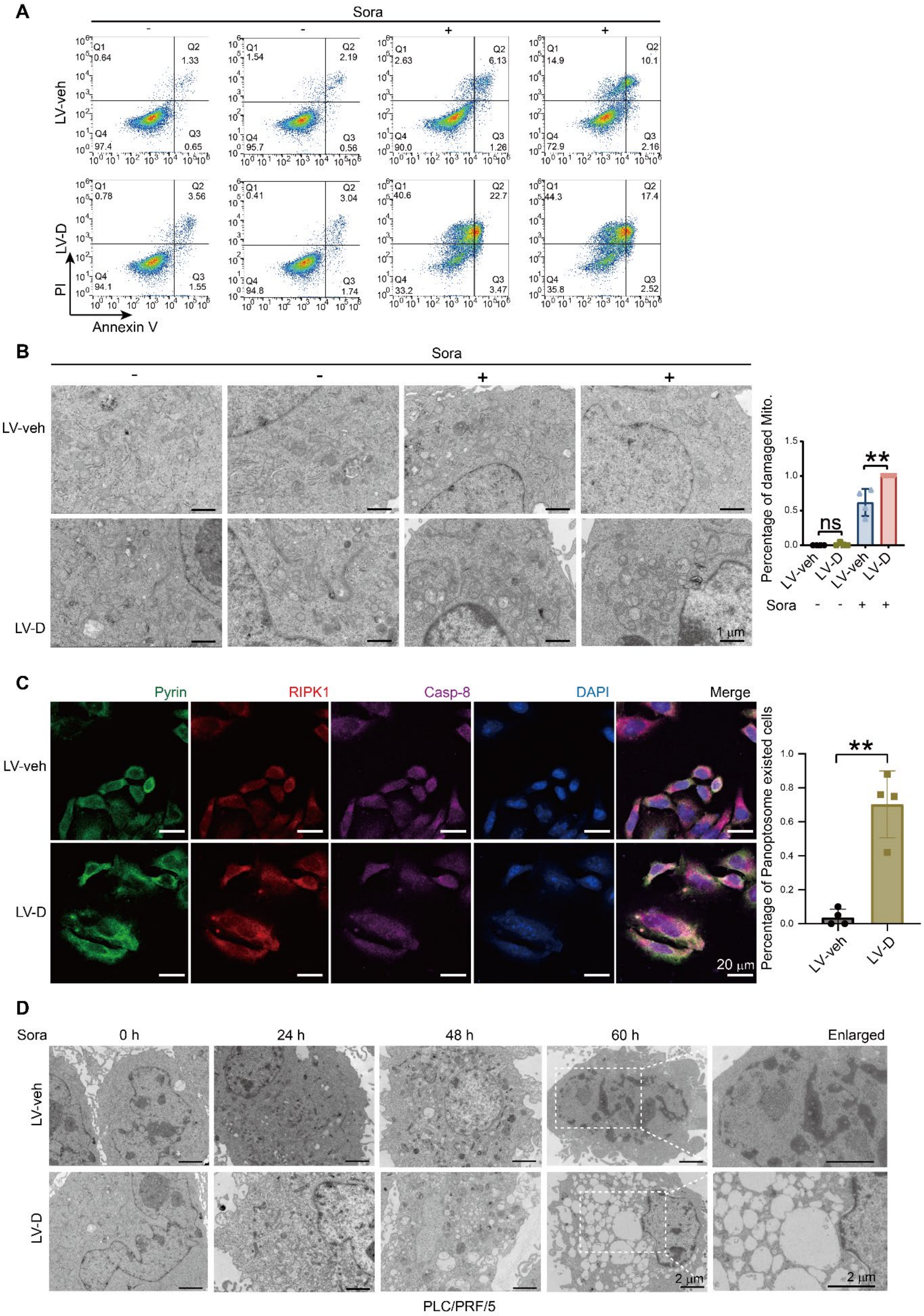
A
Sora
LV-veh
LV-D
PI
Annexin V
Q1
Q2
Q3
Q4
0.64
1.33
97.4
0.65
1.54
2.19
95.7
0.56
2.63
6.13
90.0
1.26
14.9
10.1
72.9
2.16
0.78
3.56
94.1
1.55
0.41
3.04
94.8
1.74
40.6
22.7
33.2
3.47
44.3
17.4
35.8
2.52
B
Sora
LV-veh
LV-D
1 μm
Percentage of damaged Mito.
ns
**
C
Pyrin
RIPK1
Casp-8
DAPI
Merge
LV-veh
LV-D
20 μm
Percentage of Panoptosome existed cells
**
D
Sora
0 h
24 h
48 h
60 h
Enlarged
LV-veh
LV-D
2 μm
2 μm
PLC/PRF/5

Supplementary Figure S4. DNASE1L3 promotes PANoptosis in HCC cells. (A) FCM with Annexin V/PI staining evaluating the percentages of live cells among the LV-veh and LV-D MHCC97-H HCC cells with or without sorafenib treatment (20 μM, 48 h). (**B**) Electron microscope images of LV-veh and LV-D MHCC97-H HCC cells treated with or without sorafenib treatment (20 μM, 24 h). Mitochondria were represented by orange arrows. Scale bar = 1 μm. The quantification analysis was based on the percentage of damaged mitochondria in electron microscope images. (**C**) IF assay represented Pyrin (green), RIPK1 (red), caspase-8 (purple) and DAPI (blue) in LV-veh and LV-D PLC/PRF/5 HCC cells treated with sorafenib (20 μM, 24 h). The co-expression assembled spot is PANoptosome. The quantification analysis was based on the percentage of PANoptosome existed cells in images. Scale bar = 10 μm. (**D**) Time-dependent electron microscope images of LV-veh and LV-D PLC/PRF/5 HCC cells treated with sorafenib (20 μM). Scale bar = 2 μm. The data in (**B**, **C**) are representative of four independent experiments. All the data are presented as mean ± SD. $^{**}p < 0.01$, ns represents no significant difference.

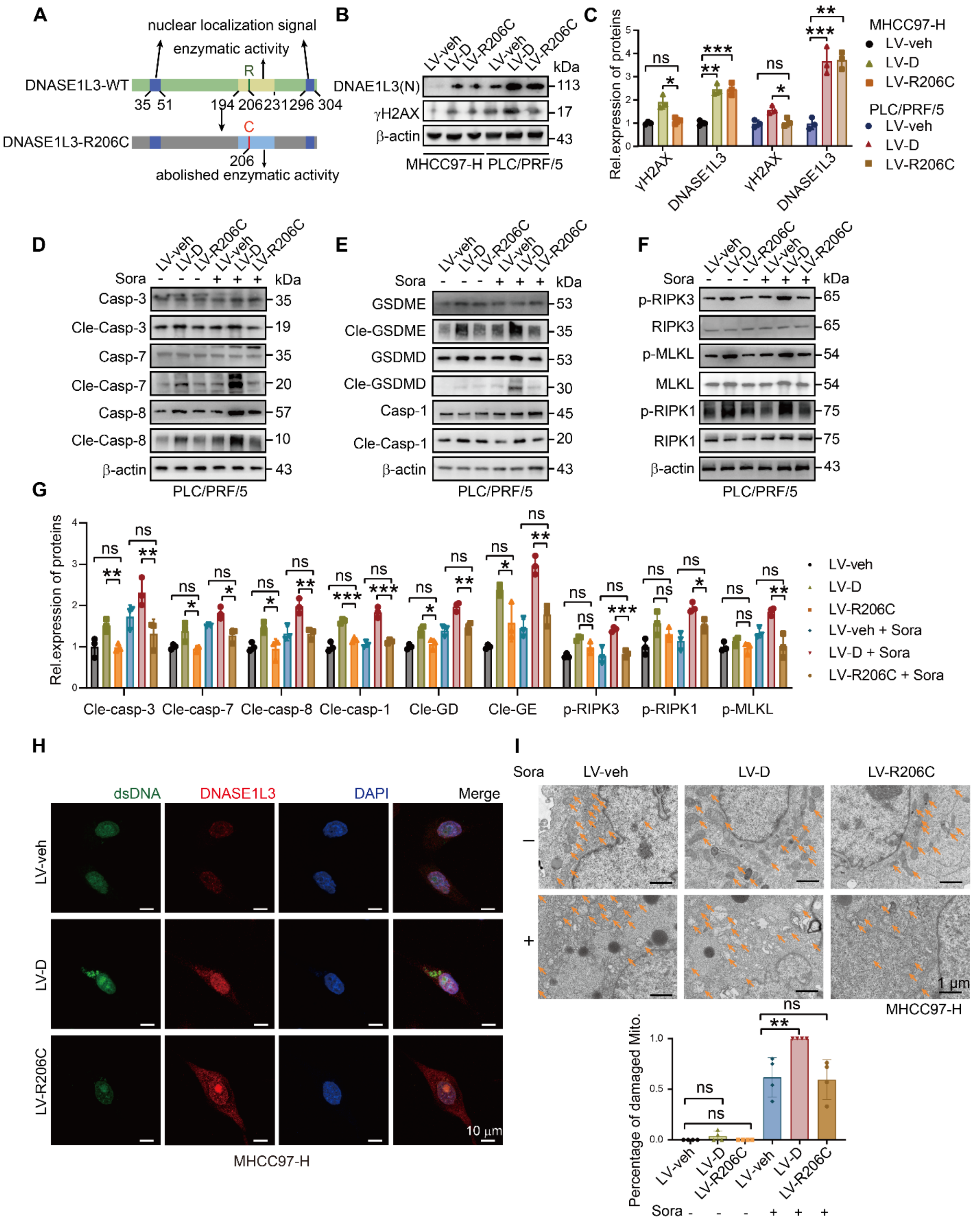

Supplementary Figure S5. DNASE1L3 induces PANoptosis through its deoxyribonuclease activity. (**A**) Schematic diagram of DNASE1L3-WT and DNASE1L3 R206C mutant. (**B**) Western

blotting analysis of DNASE1L3 and γ H2AX in LV-veh, LV-D and LV-R206C HCC cells. (**C**) Quantification of the western blotting analysis. (**D-F**) Western blotting analysis of apoptosis-related proteins (**D**), pyroptosis-related proteins (**E**), and necroptosis-related proteins (**F**) in LV-veh, LV-D and LV-R206C PLC/PRF/5 HCC cells treated with sorafenib (10 μM, 24 h). (**G**) Quantification of the western blotting analysis. (**H**) IF staining of dsDNA (green), DNASE1L3 (red) and DAPI (blue) in LV-veh, LV-D and LV-R206C MHCC97-H HCC cells treated with sorafenib (20 μM, 24 h). Scale bar = 10 μm. (**I**) Electron microscope images of LV-veh, LV-D and LV-R206C MHCC97-H HCC cells treated with sorafenib (20 μM, 24 h). Mitochondria were represented by orange arrows. Scale bar = 1 μm. The quantification analysis was based on the percentage of damaged mitochondria in electron microscope images. The data in (**C**, **G**) are representative of three independent experiments. All the data are presented as mean ± SD. $^*p < 0.05$, $^{**}p < 0.01$, $^{***}p < 0.001$, ns represents no significant difference.

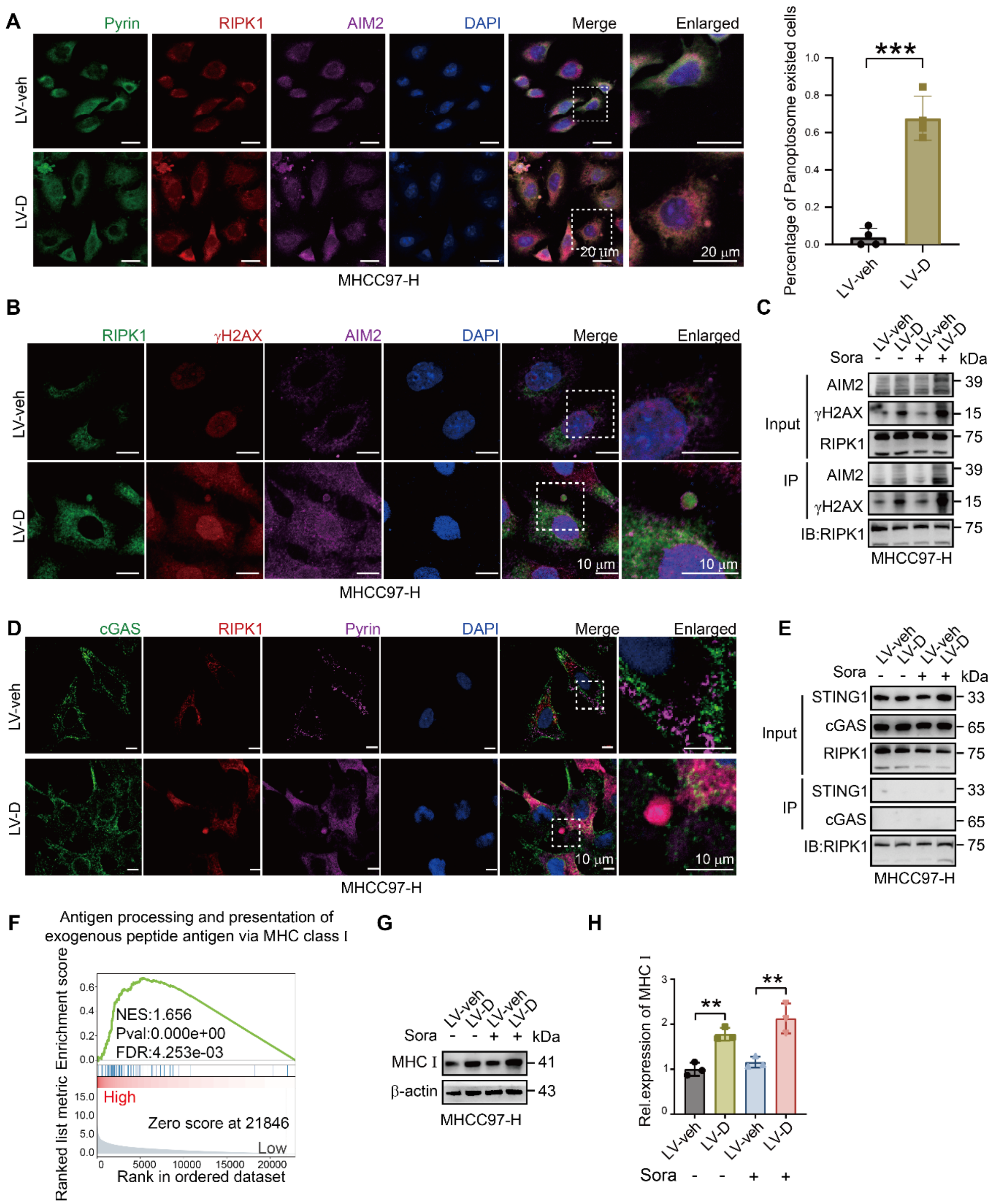

Supplementary Figure S6. DNASE1L3 induces PANoptosis through cGAS-STING independent pathway. (**A**) IF assay of Pyrin (green), RIPK1 (red), AIM2 (purple) and DAPI (blue) in LV-veh and LV-D MHCC97-H HCC cells treated with sorafenib treatment (20 μM, 24 h). The

co-expression assembled spot represents PANoptosome. Scale bar = 10 μm. The quantification analysis was based on the percentage of PANoptosome existed cells in images. (**B**) IF assay of RIPK1 (green), γH2AX (red), AIM2 (purple) and DAPI (blue) in LV-veh and LV-D MHCC97-H HCC cells treated with sorafenib treatment (20 μM, 24 h). The co-expression assembled spot represents PANoptosome. Scale bar = 10 μm. (**C**) Co-IP assay of AIM2, RIPK1 and γH2AX in LV-veh and LV-D MHCC97-H HCC cells with or without sorafenib treatment (20 μM, 24 h). (**D**) IF assay of cGAS (green), RIPK1 (red), Pyrin (purple) and DAPI (blue) in LV-veh and LV-D MHCC97-H HCC cells treated with sorafenib (20 μM, 24 h). Scale bar = 10 μm. (**E**) Co-IP assay of cGAS, STING1 and RIPK1 in LV-veh and LV-D MHCC97-H HCC cells treated with or without sorafenib treatment (20 μM, 24 h). (**F**) Representative GSEA in RNA-seq from LV-veh and LV-D MHCC97-H HCC cells. (**G**) Western blotting analysis of MHC I in LV-veh and LV-D MHCC97-H HCC cells with or without sorafenib treatment (20 μM, 24 h). (**H**) Quantification of the western blotting analysis. The data in (**A**) are representative of four independent experiments. The data in (**H**) are representative of three independent experiments. All the data are presented as mean ± SD. $^{**}p < 0.01$, $^{***}p < 0.001$.

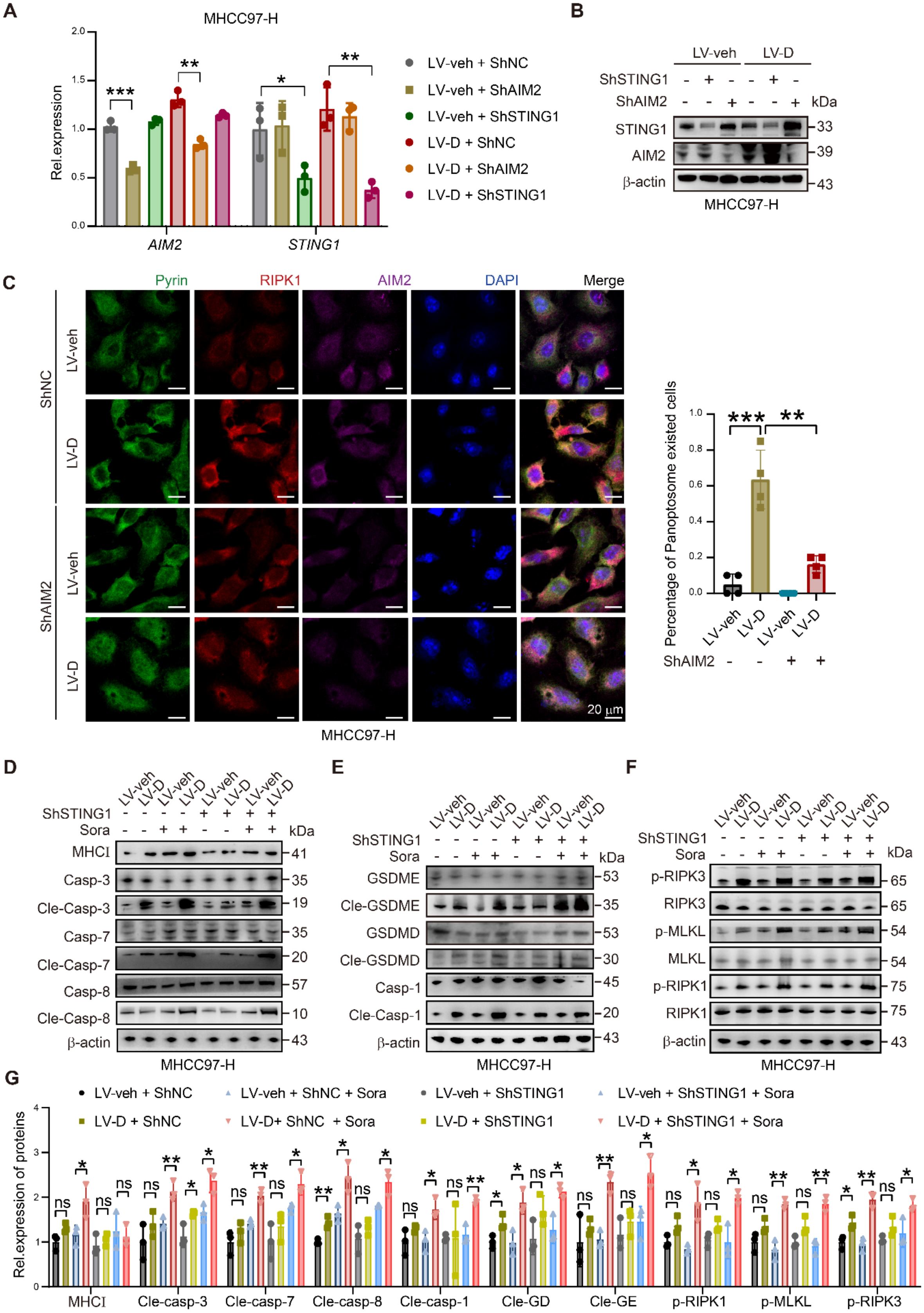
A
MHCC97-H
Rel.expression
AIM2
STING1
LV-veh + ShNC
LV-veh + ShAIM2
LV-veh + ShSTING1
LV-D + ShNC
LV-D + ShAIM2
LV-D + ShSTING1
B
LV-veh
LV-D
ShSTING1
ShAIM2
kDa
STING1
AIM2
β-actin
MHCC97-H
C
Pyrin
RIPK1
AIM2
DAPI
Merge
ShNC
ShAIM2
LV-veh
LV-D
20 μm
MHCC97-H
Percentage of Panoptosome existed cells
D
E
F
ShSTING1
Sora
MHCI
Casp-3
Cle-Casp-3
Casp-7
Cle-Casp-7
Casp-8
Cle-Casp-8
GSDME
Cle-GSDME
GSDMD
Cle-GSDMD
Casp-1
Cle-Casp-1
p-RIPK3
RIPK3
p-MLKL
MLKL
p-RIPK1
RIPK1
G
LV-veh + ShNC
LV-veh + ShNC + Sora
LV-veh + ShSTING1
LV-veh + ShSTING1 + Sora
LV-D + ShNC
LV-D+ ShNC + Sora
LV-D + ShSTING1
LV-D + ShSTING1 + Sora
Rel.expression of proteins
MHCI
Cle-casp-3
Cle-casp-7
Cle-casp-8
Cle-casp-1
Cle-GD
Cle-GE
p-RIPK1
p-MLKL
p-RIPK3

Supplementary Figure S7. DNASE1L3 induces PANoptosis through cGAS-STING independent pathway. (**A**) RT-qPCR presented the *AIM2* and *STING1* expression after the knockdown of AIM2 or STING1 in LV-veh and LV-D MHCC97-H HCC cells. (**B**) Western blotting analysis of AIM2 and STING1 in ShNC, ShAIM2, LV-veh and LV-D MHCC97-H HCC cells. (**C)** IF assay of Pyrin (green), RIPK1 (red), AIM2 (purple) and DAPI (blue) in ShNC, ShAIM2, LV-veh and LV-D MHCC97-H HCC cells treated with sorafenib (20 μM, 24 h). The co-expression assembled spot represents PANoptosome. Scale bar = 10 μm. The quantification analysis was based on the percentage of PANoptosome existed cells in images. (**D**-**E**) Western blotting analysis of MHC I as well as apoptosis-related proteins (**D**), pyroptosis-related proteins (**E**), and necroptosis-related proteins (**F**) in MHCC97-H HCC cells treated with or without sorafenib (20 μM, 24 h). (**G**) Quantification of the western blotting analysis. The data in (**A**, **G**) are representative of three independent experiments. The data in (**C**) are representative of three independent experiments. All the data are presented as mean ± SD. *p < 0.05, **p < 0.01, ***p < 0.001, ns represents no significant difference.

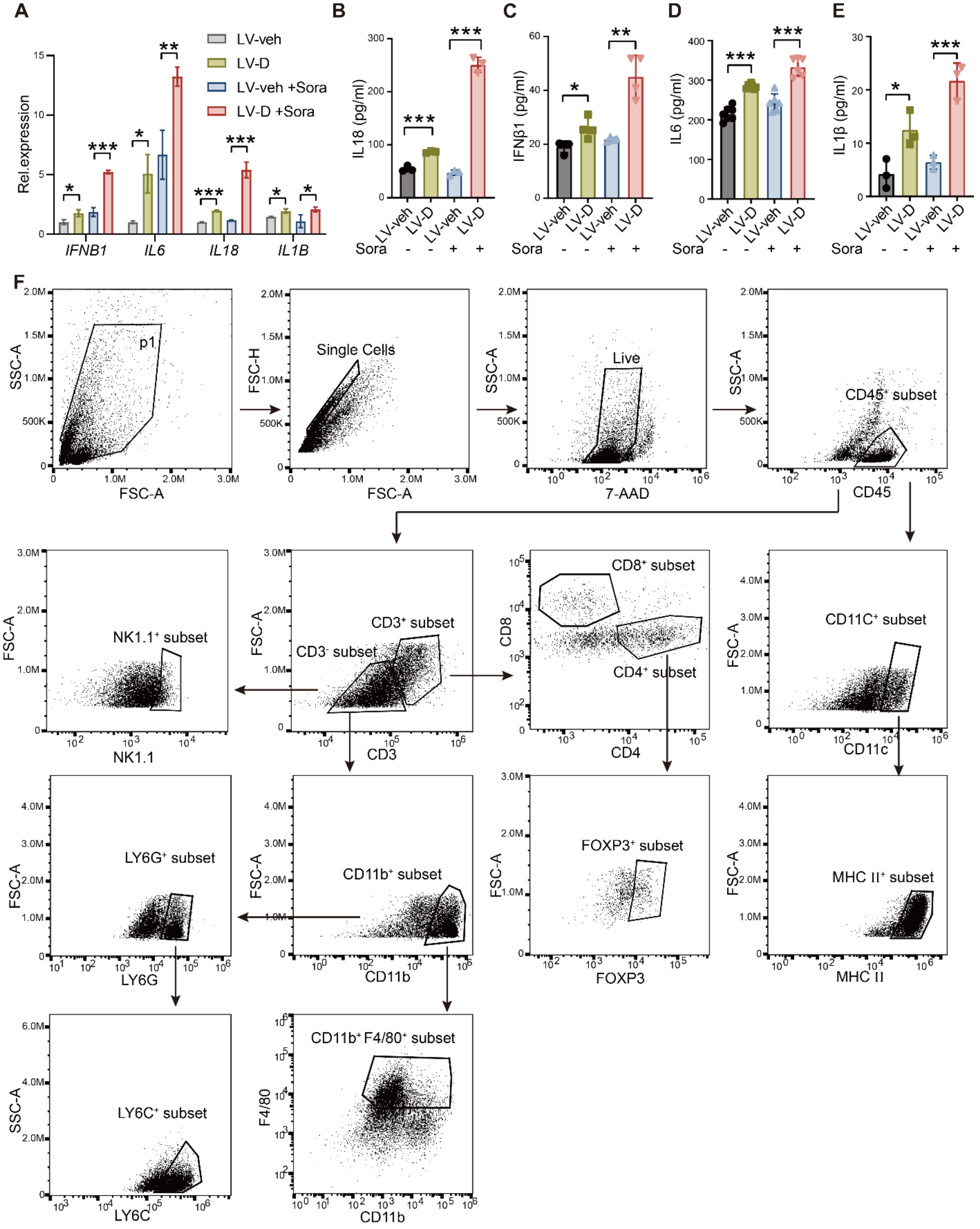

Supplementary Figure S8. DNASE1L3 promotes the production of inflammatory factors. (A)

RT-qPCR was presented the *IL18*, *IL1β*, *IL6*, *IFNβ1* expression in LV-veh and LV-D MHCC97-H HCC cells treated with or without sorafenib (20 μM, 24 h). (**B-E**) ELISA of IL18 (**B**), IFNβ1 (**C**), IL6 (**D**), IL1β (**E**) in the supernatant of LV-veh and LV-D MHCC97-H HCC cells treated with or without sorafenib (20 μM, 24 h). (**F**) The gating strategy for immune phenotyping. The data in (**A**) is representative of three independent experiments. The data in (**B**-**E**) is representative of four independent experiments. All the data are presented as mean ± SD. *$p < 0.05$, **$p < 0.01$, ***$p < 0.001$.

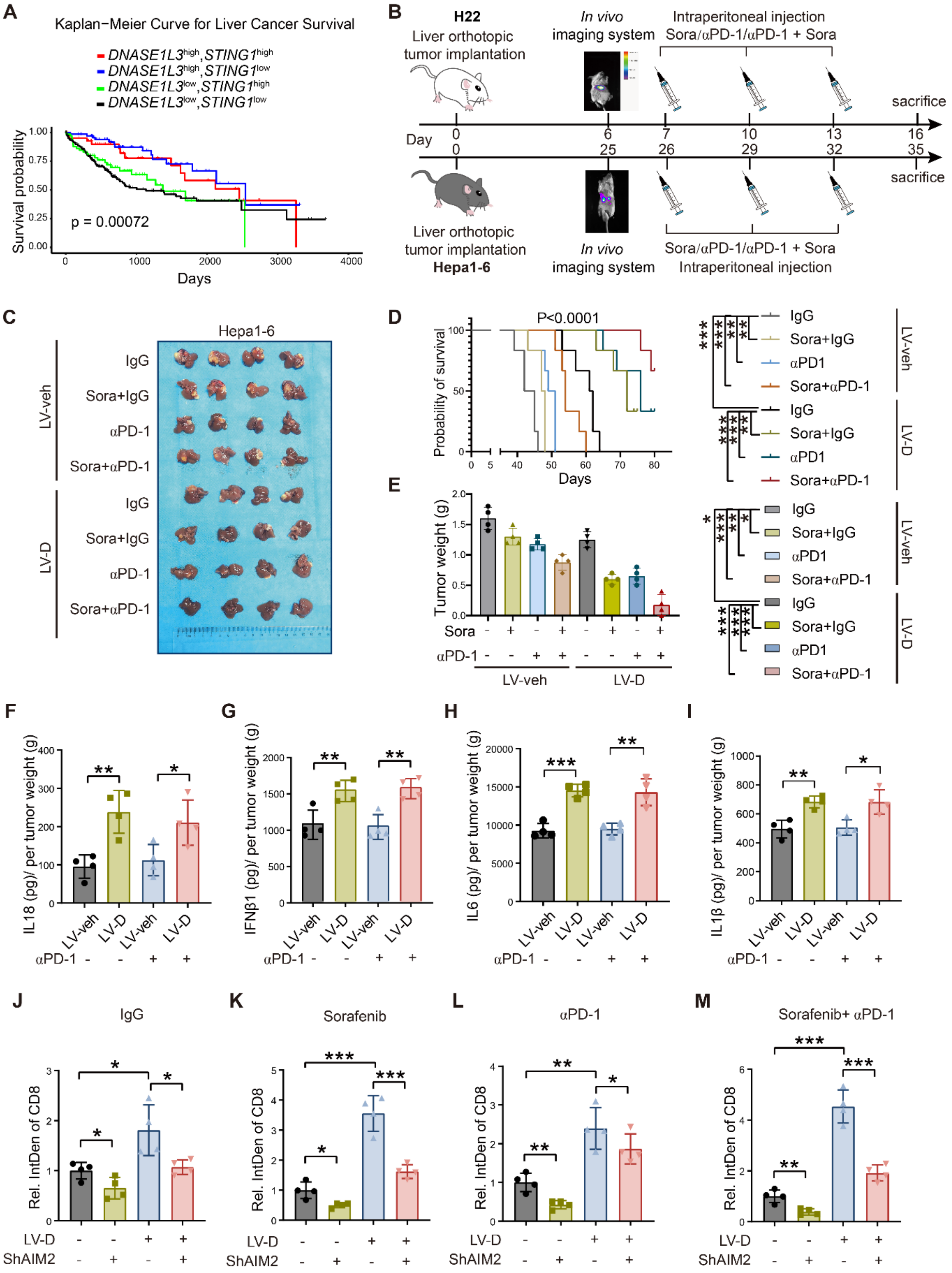
A
Kaplan−Meier Curve for Liver Cancer Survival
DNASE1L3high,STING1high
DNASE1L3high,STING1low
DNASE1L3low,STING1high
DNASE1L3low,STING1low
Survival probability
p = 0.00072
Days
B
H22
Liver orthotopic tumor implantation
In vivo imaging system
Intraperitoneal injection
Sora/αPD-1/αPD-1 + Sora
sacrifice
Day
Hepa1-6
C
Hepa1-6
LV-veh
LV-D
IgG
Sora+IgG
αPD-1
Sora+αPD-1
D
P<0.0001
Probability of survival
Days
E
Tumor weight (g)
Sora
αPD-1
LV-veh
LV-D
F
IL18 (pg)/ per tumor weight (g)
G
IFNβ1 (pg)/ per tumor weight (g)
H
IL6 (pg)/ per tumor weight (g)
I
IL1β (pg)/ per tumor weight (g)
J
IgG
K
Sorafenib
L
αPD-1
M
Sorafenib+ αPD-1
Rel. IntDen of CD8
LV-D
ShAIM2

Supplementary Figure S9. DNASE1L3 enhances the effectiveness of the combination therapy of sorafenib and αPD-1. (**A**) Kaplan-Meier survival curve based on the relative expression of *DNASE1L3* and *STING1* in liver cancer tissues from the TCGA database. (**B**) Flow diagram of constructing liver orthotopic tumor implantation mouse model. (**C**) Photographs of the excised livers from Hepa1-6 liver cancer cells constructed liver orthotopic tumor implantation mouse model treated by sorafenib monotherapy, αPD-1 monotherapy and combination therapy with sorafenib and αPD-1(n=4). (**D**) Survival curve of Hepa1-6 liver cancer cells constructed liver orthotopic tumor implantation mouse model treated by sorafenib monotherapy, αPD-1 monotherapy and combination therapy with sorafenib and αPD-1(n=6). (**E**) Statistical analysis of the tumor weight (g) of Hepa1-6 liver cancer cells constructed liver orthotopic tumor implantation mouse model treated by sorafenib monotherapy, αPD-1 monotherapy and combination therapy with sorafenib and αPD-1(n=4). (**F-I**) The mouse inflammatory factors in liver orthotopic tumor with αPD-1 treatment including IL18, IFNβ1, IL6, and IL1β were detected by ELISA. Statistical analysis was calculated per tumor weight. (**J-M**) The IntDen level of CD8 in IF in liver orthotopic tumor with non-treatment (**J**), sorafenib monotherapy (**K**), αPD-1 monotherapy (**L**), and combination therapy (**M**). The data in (**D**) is representative of six independent experiments. The data in (**E, F-M**) is representative of four independent experiments. All the data are presented as mean ± SD. $^{**}p < 0.01$, $^{***}p < 0.001$.

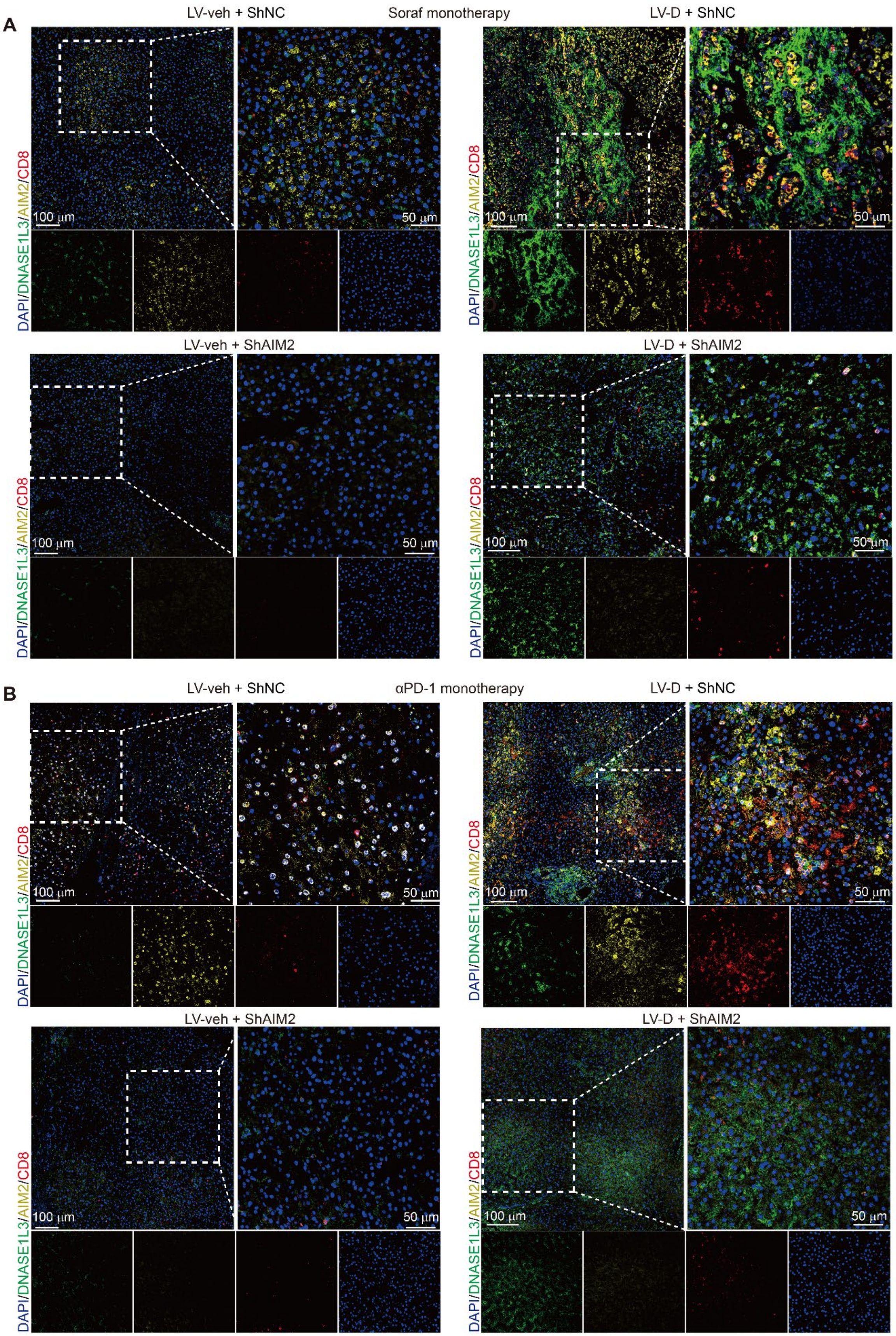

A
Soraf monotherapy
LV-veh + ShNC
LV-D + ShNC
LV-veh + ShAIM2
LV-D + ShAIM2
DAPI/DNASE1L3/AIM2/CD8
100 μm
50 μm
B
αPD-1 monotherapy
LV-veh + ShNC
LV-D + ShNC
LV-veh + ShAIM2
LV-D + ShAIM2
DAPI/DNASE1L3/AIM2/CD8
100 μm
50 μm

Supplementary Figure S10. CD8$^+$ T cells infiltration in liver orthotopic tumor mouse model with sorafenib and αPD-1 monotherapy. (**A** and **B**) IF assay was used to verify the expression of DAPI (blue), DNASE1L3 (green), AIM2 (yellow) and CD8 (red) for the H22 liver cancer cells constructed liver orthotopic tumor implantation mouse model treated by sorafenib (**A**) and αPD-1 monotherapy (**B**). Scale bars in overall images (left) is 100 μm, and in enlarged images (right) is 50 μm. Images with separated channels are below.

Table S1. Primers for RT-qPCR.

Genes (homo)	Forward primers (5' to 3')	Reverse primers (5' to 3')
DNASE1L3	GTCAGTTCTGTTGTTCCCAAGTC	CTGGAAAGTGGTCGCTGACATC
GAPDH	ACAACTTTGGTATCGTGGAAGG	GCCATCACGCCACAGTTTC
AIM2	CTGCAGTGATGAAGACCATTCGTA	GGTGCAGCACGTTGCTTTG
STING1	CCAGAGCACACTCTCCGGTA	CGCATTTGGGAGGGAGTAGTA
IL18	CTGCCACCTGCTGCAGTCTA	TCTACTGGTTCAGCAGCCATCTTTA
IL1β	CCTGTGGCCTTGGGCCTCAA	GGTGCTGATGTACCAGTTGGG
IL6	CCACACAGACAGCCACTCAC	AGGTTGTTTTCTGCCAGTGC
IFNB1	GCTTGGATTCCTACAAAGAAGCA	ATAGATGGTCAATGCGGCGTC

Table S2. DNA constructs and gene silencing.

Target Gene	Target Seq
Homo *DNASE1L3*<br>(NM_004944-**R206C**)	CDS area:<br>ATGTCACGGGAGCTGGCCCCACTGCTGCTTCTCCTCCTCTCCATCCACAGCGCCC<br>TGGCCATGAGGATCTGCTCCTTCAACGTCAGGTCCTTTGGGGAAAGCAAGCAGG<br>AAGACAAGAATGCCATGGATGTCATTGTGAAGGTCATCAAACGCTGTGACATCA<br>TACTCGTGATGGAAATCAAGGACAGCAACAACAGGATCTGCCCCATACTGATGG<br>AGAAGCTGAACAGAAATTCAAGGAGAGGCATAACGTACAACTATGTGATTAGC<br>TCTCGGCTTGGAAGAAACACATATAAAGAACAATATGCCTTTCTCTACAAGGAA<br>AAGCTGGTGTCTGTGAAGAGGAGTTATCACTACCATGACTATCAGGATGGAGAC<br>GCAGATGTGTTTTCCAGGGAGCCCTTTGTGGTCTGGTTCCAATCTCCCCACACTG<br>CTGTCAAAGACTTCGTGATTATCCCCCTGCACACCACCCCAGAGACATCCGTTA<br>AGGAGATCGATGAGTTGGTTGAGGTCTACACGGACGTGAAACACCGCTGGAAG<br>GCGGAGAATTTCATTTTCATGGGTGACTTCAATGCCGGCTGCAGCTACGTCCCC<br>AAGAAGGCCTGGAAGAACATCTGCTTGAGGACTGACCCCAGGTTTGTTTGGCTG<br>ATCGGGGACCAAGAGGACACCACGGTGAAGAAGAGCACCAACTGTGCATATGA<br>CAGGATTGTGCTTAGAGGACAAGAAATCGTCAGTTCTGTTGTTCCCAAGTCAAA<br>CAGTGTTTTTGACTTCCAGAAAGCTTACAAGCTGACTGAAGAGGAGGCCCTGGA<br>TGTCAGCGACCACTTTCCAGTTGAATTTAAACTACAGTCTTCAAGGGCCTTCACC<br>AACAGCAAAAAATCTGTCACTCTAAGGAAGAAAACAAAGAGCAAACGCTCCTA<br>G
Homo *AIM2*	GCCACTAAGTCAAGCTGAAAT
Homo *STING1*	GTCCAGGACTTGACATCTTAA
Homo *DNASE1L3*	GGACAAGAAATCGTCAGTT
Mus *AIM2*	CTAACCACGAAGTCCCAAATA

Table S3. Clinical characteristics of participants.

Groups	No.	Age group	HBV (Pos/Neg)	Primary tumor Size (cm×cm×cm)	Time to recurrence (month)	Treatment	RESIST 1.1	AFP (ng/mL)	Baseline tumor load after recurrence (cm)
Combination therapy response	01	50s	Positive	2.5×3×2	11M	Sorafenib + Sintilimab	PR	623.1	3.6×2.8
	02	80s	Positive	15×13×7	14M	Sorafenib + Camrelizumab	SD	1.28	6.5×5.5
	03	50s	Positive	2.6×2.5×2.5	6M	Sorafenib + Sintilimab	SD	>2000	1.9×2.0
	04	30s	Positive	1×0.6×0.2	2M	Sorafenib + Camrelizumab	SD	>2000	8.3×5.8
Combination therapy poor-response	05	40s	Positive	2×1.8×1.7	19M	Sorafenib + Sintilimab	PD	159.77	2.5×5.7
	06	60s	Negative	6×2×1.5	3M	Sorafenib + Camrelizumab	PD	>2000	R = 1.2
	07	40s	Positive	12×10.5×10.5	15M	Sorafenib + Camrelizumab	PD	3.03	R = 3.7
	08	40s	Positive	6×5×5	4M	Sorafenib + Camrelizumab	PD	>2000	10×7.5
Sorafenib monotherapy response	09	70s	Positive	15×13×8	2M	sorafenib	PR	5.78	2.4×2.1
	10	30s	Positive	6×5×4	12M	sorafenib	SD	22.44	R = 2.3
	11	50s	Positive	3×3×2.5	11M	sorafenib	PR	3.96	5×3.8
	12	50s	Positive	3.5×3×2.2	15M	sorafenib	PR	3.09	4.2×4.2
Sorafenib monotherapy poor-response	13	50s	Positive	19×10×8	2M	sorafenib	PD	15.78	R = 3.4
	14	50s	Positive	5.5×2.6×1.6	18M	sorafenib	PD	2.95	R = 3
	15	60s	Positive	2.5×2×1.2	13M	sorafenib	PD	3.84	0.8×1.1
	16	50s	Positive	1.5×1.5×1	24M	sorafenib	PD	8.23	R =0.5
HCC patients	17	40s	Positive	6.5×6.5×6	N/A	N/A	N/A	600.88	N/A
	18	40s	Negative	15×12×6	N/A	N/A	N/A	4.55	N/A
	19	40s	Positive	3×3×3	N/A	N/A	N/A	10.15	N/A
	20	40s	Positive	3×2×1	N/A	N/A	N/A	70.56	N/A
	21	50s	Positive	0.1×2×0.2	N/A	N/A	N/A	444.48	N/A
	22	60s	Positive	2×2×1.5	N/A	N/A	N/A	2.69	N/A
	23	50s	Positive	9.3×9×8	N/A	N/A	N/A	225.69	N/A
	24	30s	Positive	1.6×1.5×1.5	N/A	N/A	N/A	>2000	N/A
	25	40s	Positive	5.5×4×3.5	N/A	N/A	N/A	107.48	N/A
	26	60s	Positive	4.5×4.5×3.5	N/A	N/A	N/A	>2000	N/A
	27	50s	Positive	4.5×4×3.5	N/A	N/A	N/A	402.88	N/A
	28	60s	Positive	5×3×3	N/A	N/A	N/A	2.39	N/A

(N/A= none)

Materials and Methods for

DNASE1L3-mediated PANoptosis enhances the efficacy of combination therapy for advanced hepatocellular carcinoma

Jingchun Wang[1], Yu Chen[2], Yanquan Xu[3], Jiangang Zhang[2], Shuai Yang[4], Yu Zhou[2], Juan Lei[2], Ran Ren[2], Yang Chen[2], Huakan Zhao[2]*, Yongsheng Li[2]*, Shiming Yang[1]*

***Corresponding author emails**: (lys@cqu.edu.cn) (Y.L.), (hk_zhao@cqu.edu.cn) (H.Z.), and (yangshiming@tmmu.edu.cn) (S.Y.)

This PDF file includes:

Materials and Methods

Materials and Methods

Analysis of differentially expressed genes

We downloaded raw RNA-seq counts from 424 TCGA-LIHC samples via the GDC data portal and conducted our analysis based on low (n = 100) and high (n = 100) expression levels of DNASE1L3. DEGs were identified using edgeR, with the analysis threshold set at $-0.5 \geqslant \log_2$[fold change] $\geqslant 0.5$ between samples with low and high DNASE1L3 expression. Genes exhibiting a p-value < 0.05 were classified as differentially expressed. Subsequently, a heatmap illustrating gene z-scores was generated using R, highlighting genes associated with immune signatures. For GO analysis, all DEGs were submitted to the Gene Ontology database (http://www.geneontology.org/) for term mapping, followed by the calculation of the number of genes linked to each term. Statistical methods, including hypergeometric testing, were employed to compare these genes against the entire genomic background. The enriched GO data were filtered, applying a significance threshold of $p < 0.05$.

Gene set enrichment analysis (GSEA) and ingenuity pathway analysis (IPA)

GSEA was conducted using the GSEA Desktop Application, which can be downloaded from software.broadinstitute.org/gsea/. The analysis utilized gene expression data from human liver cancer specimens acquired from TCGA to identify genes that exhibited differential expression between patient groups characterized by specific traits, such as low versus high expression of *DNASE1L3*. The results included high-ranked gene sets along with enrichment plots, normalized enrichment scores (NES), nominal p-values, and false discovery rate (FDR-q) values. Additionally, heatmap plots were generated using FPKM values for DNASE1L3 low (n = 100) and high (n = 100) expression groups from TCGA-LIHC samples for each gene set, including Hallmark and GO.

These FPKM values were log2 transformed (FPKM + 0.1) and subsequently converted to row (gene) z-scores using the scale function in R. All analyses were conducted using R version 3.6.1. For IPA, differentially expressed genes with an unpaired t-test p-value of < 0.05 between the low and high expression groups were subjected to core analysis. The results included high-ranked canonical pathways along with p-values (calculated using a right-tailed Fisher's exact test), ratios (indicating pathway coverage), and Z-scores that reflect pathway directionality, represented by filled blue bars.

Survival analysis

We conducted an analysis of the expression levels of DNASE1L3 in our samples, correlating these levels with the corresponding survival time data from the TCGA dataset. The survival analysis was performed using the survival package in R. Furthermore, we executed a combined survival analysis that considered the expression of *DNASE1L3* alongside *AIM2* or *STING1*, utilizing the same survival package in R for this purpose. For the survival analysis of DNASE1L3 expression in the context of HCC treatment, we employed the online platform available at https://kmplot.com/analysis/, specifically selecting sorafenib treatment and immunotherapy to generate the Kaplan-Meier plot.

***DNASE1L3* expression in various pathological HCC stages**

The statistics were derived from the Wurmbach liver dataset. The four pathological stages of hepatocellular carcinoma (HCC) were defined as follows: (i) very early HCC, characterized by well-differentiated tumors less than 2 cm in diameter, with no evidence of vascular invasion or satellite lesions (size range: 8-20 mm); (ii) early HCC, encompassing tumors measuring less than

2 cm that exhibit microscopic vascular invasion or satellite lesions, as well as well- to moderately differentiated tumors ranging from 2 to 5 cm that do not show vascular invasion or satellite lesions, or two to three well-differentiated nodules each measuring less than 3 cm (size range: 3-45 mm); (iii) advanced HCC, which includes poorly differentiated tumors measuring less than 2 cm with microvascular invasion or satellite lesions, or tumors measuring less than 5 cm; and (iv) very advanced HCC, defined by the presence of macrovascular invasion or diffuse involvement of the liver.

Immunohistochemistry (IHC)

Immediately after removal from the mice, the subcutaneous tumors were placed in 10% neutral-buffered formaldehyde for a minimum of 24 h. Subsequently, hematoxylin and eosin (H&E) staining, along with DNASE1L3 (CAS#ab203669, Abcam, UK, 1:100 dilution) and IgG as a negative control, was performed on 4 mm sections. Detection of the sections was carried out using fluorescence scanning microscopy (Olympus, Japan).

Immunofluorescence (IF)

Tyramide signal amplification (TSA, CAS#G1236-50T, Servicebio, China) was employed to enhance IF staining in paraffin-embedded tumor sections. The sections were prepared for IF by undergoing rehydration and antigen retrieval, following the established IHC protocol. Prior to blocking with BSA, the sections were incubated in 3% hydrogen peroxide (H_2O_2) for 30 minutes, ensuring protection from light exposure. Primary antibodies including human DNASE1L3 (CAS#ab152118, Abcam, UK, 1:500 dilution), human CD45 (CAS#ab40763, Abcam, UK, 1:2000 dilution), human CD8 (CAS#ab237709, Abcam, UK, 1:500 dilution), human Pyrin (CAS#24280-

1-AP, Proteintech, China, 1:1000 dilution), human RIPK1 (CAS# 3493S, Cell Signaling Technology, USA, 1:1000 dilution), human caspase-8 (CAS#ab308013, Abcam, UK, 1:1000 dilution), human IRF3 (CAS#11904S, Cell Signaling Technology, USA, 1:1000 dilution), human AIM2 (CAS#ab204995, Abcam, UK, 1:1000 dilution), human ASC (CAS#ab283684, Abcam, UK, 1:1000 dilution), human γH2AX (CAS#ab283684, Abcam, UK, 1:1000 dilution), mouse DNASE1L3 (CAS#67041-1-Ig, Proteintech, China, 1:200 dilution), mouse CD8 (CAS#ab217344, Abcam, UK, 1:500 dilution), and mouse AIM2 (CAS#66902-1-Ig, Proteintech, China, 1:500 dilution) were incubated with the sections overnight at 4°C, protected from light. The sections were then stained with corresponding horseradish peroxidase (HRP) secondary antibodies (OriGene, China, 1:500 dilution) for 1 h. After washing, the sections were incubated in Alexa Fluor 488 Tyramide for 10 minutes. Subsequently, the sections were soaked in antigen retrieval liquid and processed in a microwave at 95°C for 20 minutes. The second set of primary antibodies were stained in the same manner. The slides were counterstained with DAPI (CAS#C1006, Beyotime, China) and sealed with antifade solution (CAS#P0126-25ml, Beyotime, China). The IF-stained slides were visualized using a fluorescence microscope aided by Slidebook software and further assessed using a scanning system. The ratio of integrity density (IntDen) was calculated by dividing the area containing DNASE1L3, $CD45^+$, or $CD8^+$ by the area of $DAPI^+$ (Image J, DEU).

Flow Cytometry

Ki-67 (CAS#350510, Biolegend, UK, 1:100 dilution) was utilized to detect cell proliferation, while Annexin V/PI (CAS#556570, BD, USA, 1:100 dilution) was used to detect cell apoptosis induced by sorafenib. To measure cellular lipid ROS levels, the BODIPY™ 581/591 C11 probe

(CAS#D3861, Thermo Scientific, USA, 1:1000 dilution) was added, and the mixture was incubated in the dark at 37 °C for 50 minutes. For immune phenotyping, Hepa1-6 tumors were collected and processed using a gentleMACS™ C Tube (CAS#130-093-237, Miltenyi Biotec, DEU). Collagenase type IV (CAS#17104019, Thermo Scientific, USA, 5 mg), Collagenase type I (CAS#17100017, Thermo Scientific, USA, 5 mg) were added to the tube containing 5 mL DMEM. The tube was then run on program 37C_m_LIDK_1 in the gentleMACS Dissociator (CAS#130-093-235, Miltenyi Biotec, DEU) for 30 minutes. Single cells for flow cytometry were prepared using a tube-secured cell strainer with 70 μm pore size (CAS#352350, Falcon, USA). Total cells were counted, and cells in FACS staining buffer (CAS#420508, Biolegend, UK,) were treated with the following sets of antibodies: cocktail 1 [L/D (eBioscience™ Fixable Viability Dye eFluor™ 780, CAS#65-0865-14, Thermo Scientific, USA, 1:1000 dilution), Brilliant Violet 510™ anti-mouse CD45 Antibody (CAS# 103138, Biolegend, UK, 1:100 dilution), FITC anti-mouse CD3 Antibody (CAS#100204, Biolegend, UK, 1:100 dilution), PE anti-mouse NK-1.1 Antibody (CAS# 156504, Biolegend, UK, 1:100 dilution), PE/Cyanine7 anti-mouse CD4 Antibody (CAS# 100422, Biolegend, UK, 1:100 dilution), Brilliant Violet 421™ anti-mouse CD8a Antibody (CAS# 100738, Biolegend, UK, 1:100 dilution), and APC anti-mouse Foxp3 Antibody (APC, CAS# 17-5773-82, Thermo Scientific, USA, 1:100 dilution)], and cocktail 2 [L/D (eBioscience™ Fixable Viability Dye eFluor™ 780), Brilliant Violet 510™ anti-mouse CD45 Antibody, PE anti-mouse/human CD11b Antibody (CAS# 101208, Biolegend, UK, 1:100 dilution), Brilliant Violet 421™ anti-mouse CD11c Antibody (CAS# 117329, Biolegend, UK, 1:100 dilution), APC anti-mouse F4/80 Antibody (CAS# 123116, Biolegend, UK, 1:100 dilution), PerCP anti-mouse I-A/I-E Antibody (CAS# 107624, Biolegend, UK, 1:100 dilution), AF700 anti-mouse Ly6C Antibody (CAS# 56-9668-82, Thermo Scientific, USA, 1:100 dilution), Brilliant Violet 605™ anti-mouse Ly-6G

Antibody (CAS# 127639, Biolegend, UK, 1:100 dilution)]. The stained cells were fixed in 1% formaldehyde in PBS (pH 7.4) for 30 minutes at 4°C and analyzed using a flow cytometer (CytoFLEX, Beckman Coulter, USA). For intranuclear staining of Foxp3, cells were fixed and permeabilized using a FoxP3/Transcription factor Fixation/Permeabilization kit (CAS# 00-5523-00, Thermo Scientific, USA). The number of specific immune cells per gram of tumor was calculated based on the percentage of specified immune cells identified in FACS. The gating strategy for immune phenotyping is provided in Supplementary Fig. 7F.

CCK8 assay

The IC_{50} analysis, cell proliferation, and cell viability assessments were performed using the CCK8 kit (CAS#CK04, Dojindo Laboratories, Japan), in accordance with the manufacturer's instructions. For the cell proliferation experiments, the cells were resuspended to a concentration of 2×10^4 cells/mL. Subsequently, 100 μL of this cell suspension was inoculated into each well of a 96-well plate, which included experimental, blank, and control wells. The plate was incubated overnight at 37 °C. Samples were evaluated every 24 h over a period of five consecutive days. For detection, the supernatant from each well was carefully removed, and 100 μL of CCK-8 working solution (diluted at a ratio of 1:10) was added to each well, followed by a 2 h incubation period. The optical density (OD) was then measured at 450 nm using a Varioskan Flash Spectral Scanning Multimode Reader (Thermo Scientific, USA).

In the IC_{50} experiments, the experimental cells were resuspended to a concentration of 1×10^5 cells/mL and inoculated into a 96-well plate, with 100 μL dispensed into each well. The cells were incubated overnight at 37 °C. Sorafenib was diluted to various concentrations, which were subsequently added to each well and incubated for 48 h. For MHCC97-H cells, the concentrations

of sorafenib utilized were 0, 5, 10, 20, 30, 40, 50, and 60 μM. For PLC/PRF/5 cells, the concentrations were 0, 2, 3, 4, 8, 10, 15, 20, 30, and 40 μM. For HepG2 cells, the concentrations ranged from 0.5 to 32 μM, specifically 0.5, 1, 2, 4, 8, 12, 16, 20, and 32 μM. All concentrations were determined based on preliminary experiments. Following the 48h sorafenib treatment, the supernatant in each well was removed, and 100 μL of CCK-8 working solution (diluted 1:10) was added, followed by a 2h incubation period. The optical density (OD) was measured at 450 nm using a Varioskan Flash Spectral Scanning Multimode Reader (Thermo Scientific, USA).

For the assessment of cell viability, experimental cells were resuspended to a concentration of 1×10^5 cells/mL and inoculated into a 96-well plate, with 100 μL added to each well. The cells were incubated overnight at 37 °C. Apoptotic (CAS#HY-16658B, MedChemExpress, USA), necroptotic (CAS#HY-15760, MedChemExpress, USA), and pyroptotic (CAS#HY-111675, MedChemExpress, USA) inhibitors were pre-incubated for 2 h, followed by treatment with sorafenib for 48 h under the same conditions. The absorbance at 450 nm was measured using the Varioskan Flash Spectral Scanning Multimode Reader (Thermo Scientific, USA).

For the cell viability assay using LV-D cell culture supernatant, we collected the supernatant after 48 h of incubation. The experimental cells were resuspended to a concentration of 1×10^5 cells/mL and inoculated into a 96-well plate, with 100 μL added to each well, followed by overnight incubation at 37 °C. Subsequently, we removed the culture medium and replaced it with the LV-D cell culture supernatant containing sorafenib, which was administered for an additional 48 h. The remaining steps were conducted as previously described. For the cell viability assay involving the DNASE1L3 recombination protein (CAS#HY-P71543, MedChemExpress, USA), we added the protein at a concentration of 2 ng/mL in conjunction with sorafenib treatment. The subsequent steps were consistent with those outlined earlier. Each sample in the CCK-8 assay

consisted of five replicate wells, and each experiment was conducted independently three times or more.

Cell toxicity

The lactate assay (CAS#L256, Dojindo Laboratories, Japan) was employed to evaluate cell toxicity. A suspension of 1×10^5 cells/mL was inoculated into a 96-well plate, with 100 μL added to each well, and incubated overnight at 37 °C. Various inhibitors were pre-incubated for 2 h prior to treatment with sorafenib for a duration of 48 h. Following this, the cell supernatant was collected to determine the lactate concentration, adhering to the manufacturer's instructions (Dojindo Laboratories, Japan).

TdT-mediated dUTP nick-end labeling (Tunel) assay

The DAB (SA-HRP) TUNEL Cell Apoptosis Detection Kit (CAS# T2190, Solarbio Life Science, China) was employed for the TUNEL assay. In summary, paraffin-embedded sections underwent deparaffinization, rehydration, and permeabilization, followed by a blocking step with 3% H_2O_2 for 20 minutes to inhibit endogenous peroxidase activity. Subsequently, the sections were incubated with TdT reaction cocktails for 60 minutes at 37 °C and treated with a streptavidin-HRP (OriGene, China, 1:100 dilution) reaction mixture for an additional 30 minutes at 37 °C. Staining images were captured using fluorescence scanning microscopy (Olympus, Japan) to quantify the Integrated Density (IntDen) of TUNEL-positive cells using ImageJ (DEU).

Transmission electron microscope

The cell resuspension solution was subjected to centrifugation at 1300 r/min. The resulting sediment was promptly fixed with 2.5% glutaraldehyde in 0.1 mol/L sodium cacodylate and subsequently stored at 4°C until embedding. Following this, the samples underwent postfixation with 1% osmium tetroxide and were dehydrated using a gradient of increasing concentrations of ethanol and propylene oxide. Ultrathin sections, measuring 50-60 nm, were then prepared from the embedded samples using an ultramicrotome. Finally, the sections were stained with 3% uranyl acetate and lead citrate before being examined using a JEM-1200 electron microscope (JEOL, Japan) at an accelerating voltage of 80 kV.

Seahorse analysis

The Seahorse Analyzer XF96 (Agilent, USA) was employed to assess the oxygen consumption rate (OCR) of various HCC cell lines, adhering to the manufacturer's protocols. Specifically, 5×10^3 cells per well were cultured in XF96 cell culture plates using DMEM medium. The XF calibrator was introduced to each well of the XF cartridge and incubated overnight at 37°C. For the Cell Mito Stress Test, the Seahorse XF Base Medium (CAS#102334-100, Agilent, USA), supplemented with 1 mM pyruvate (CAS#103578, Agilent, USA), 2 mM glutamine (CAS#103579, Agilent, USA), and 10 mM glucose (CAS#103577, Agilent, USA), was pre-warmed to 37°C. This warmed medium was utilized to wash the cells, followed by the addition of 180 µL per well. The Seahorse XFp Mito Stress Test Kit (CAS#103010-100, Agilent, USA) was utilized in accordance with the manufacturer's instructions. The plates were then incubated at 37°C for approximately 40 minutes before the OCR measurements were obtained using the Seahorse XFp Instrument (Agilent, USA).

Quantification of ATP

The concentration of ATP levels was determined using ATP Assay kits (CAS#S0026, Beyotime, China) and measured via spectrophotometric analysis. Specifically, experimental cells were resuspended in 6-well plates and incubated overnight at 37 °C. Following incubation, the culture medium was aspirated, and a lysate was added at a ratio of 200 μl per well to lyse the cells. After lysis, the cells were centrifuged at 12,000g for 5 minutes at 4°C, and the supernatant was collected for subsequent measurement. For detection, 100 μl of ATP Assay Working Solution was added to each well of a 96-well assay plate and allowed to stand at room temperature for 3-5 minutes to ensure complete consumption of background ATP. Subsequently, 20 μl of samples were added to each well and mixed rapidly. The results were then detected using the Varioskan Flash Spectral Scanning Multimode Reader (Thermo Scientific, USA). Finally, the results were normalized to the total protein concentration of the cells.

Immunoblotting and Immunoprecipitation

Immunoblotting was conducted following standard protocols. HCC cells were lysed by incubation in RIPA buffer (CAS# P0013B, Beyotime, China) and subjected to ultrasonication for 30 seconds. For immunoprecipitation, HCC cells were lysed using a 1% Triton X-100 buffer (CAS# ST797-500ml, Beyotime, China) and underwent three freeze-thaw cycles. The resulting lysates were incubated overnight with the specified antibodies and subsequently with protein A/G agarose beads (CAS# P2195M, Beyotime, China) for 4 h under gentle mixing. Proteins were eluted by the addition of lysis buffer and boiled in Laemmli buffer prior to separation via SDS-PAGE (CAS# P0012AC, Beyotime, China) and transfer to a PVDF membrane (CAS# FFP19, Beyotime, China). The membranes were incubated for 1 h at room temperature with a blocking solution (CAS#

P0023B-500ml, Beyotime, China), followed by overnight incubation at 4°C with the appropriate primary antibodies. Afterward, the membranes were washed with TBS and incubated for 1 h at room temperature with an HRP-conjugated secondary antibody (OriGene, China, 1:100 dilution). Blots were treated with a chemiluminescent substrate (CAS# A38556, Thermo Scientific, USA), and protein bands were visualized and quantified using a ChemiDoc Imaging System (Bio-Rad, USA). The following antibodies were used to detect proteins or tags: DNASE1L3 (CAS#ab152118, Abcam, UK, 1:1000 dilution), β-actin (CAS#81115-1-RR, Proteintech, China, 1:5000 dilution), caspase-3 (CAS#9662S, Cell Signaling Technology, USA, 1:1000 dilution), caspase-7 (CAS# 9492S, Cell Signaling Technology, USA, 1:1000 dilution), caspase-8(CAS# 9746S, Cell Signaling Technology, USA, 1:1000 dilution), GSDME (CAS# ab215191, Abcam, UK, 1:500 dilution), cleaved N-terminal GSDME (CAS# ab222408, Abcam, UK, 1:500 dilution), GSDMD (CAS#ab215203, Abcam, UK, 1:500 dilution), cleaved C-terminal GSDMD (CAS# ab227821, Abcam, UK, 1:500 dilution), caspase-1 (CAS#ab207802, Abcam, UK, 1:500 dilution), p-RIPK3 (CAS# 93654S, Cell Signaling Technology, USA, 1:500 dilution), RIPK3(CAS# 10188S, Cell Signaling Technology, USA, 1:1000 dilution), p-MLKL (CAS#ab187091, Abcam, UK, 1:500 dilution), MLKL (CAS#ab184718, Abcam, UK, 1:1000 dilution), p-RIPK1 (CAS# 96323SF, Cell Signaling Technology, USA, 1:500 dilution), RIPK1 (CAS# 3493S, Cell Signaling Technology, USA, 1:1000 dilution), AIM2 (CAS#ab204995, Abcam, UK, 1:1000 dilution), Pyrin (CAS#24280-1-AP, Proteintech, China, 1:1000 dilution), γH2AX (CAS#ab283684, Abcam, UK, 1:1000 dilution), DNASE1L3(N) (CAS#PA5-107113, Thermo Scientific , USA, 1:500 dilution), p-IRF3 (CAS# 37829S, Cell Signaling Technology, USA, 1:1000 dilution), IRF3 (CAS# 4302S, Cell Signaling Technology, USA, 1:1000 dilution), p-TBK1 (CAS# 5483S, Cell Signaling Technology, USA, 1:1000 dilution), TBK1 (CAS# 3504S, Cell Signaling Technology, USA,

1:1000 dilution), p-STING (CAS# 50907S, Cell Signaling Technology, USA, 1:500 dilution), STING (CAS# 13647S, Cell Signaling Technology, USA, 1:500 dilution), MHCI (CAS#ab134189, Abcam, UK, 1:1000 dilution).

Caspase-1 and caspase-3 enzyme activity

Caspase-1 (CAS# KTA3020, Abbkine, China) and caspase-3 (CAS# KTA3022, Abbkine, China) enzyme activities were assessed using an enzyme activity analysis kit, in accordance with the manufacturer's instructions. The resulting enzyme activity values were subsequently normalized to the total protein concentration of the cells.

Whole-transcriptome sequencing (RNA seq)

We sent LV-veh and LV-D MHCC97-H cells (n=4) to Novogene (China) for RNA sequencing. The sequencing libraries were prepared using the NEBNext® UltraTM RNA Library Prep Kit for Illumina® (NEB, USA), adhering strictly to the manufacturer's guidelines. Index codes were incorporated to assign sequences to each sample (Illumina). GO enrichment analysis was conducted utilizing the clusterProfiler R package, while the statistical significance of differentially expressed genes was assessed through KEGG pathway analysis.

DsDNA staining

The dsDNA in HCC cells was stained using the Picogreen dsDNA assay kit (CAS#MF0781-1ML, MKBio, China). The cells were cultured on slides overnight before being incubated with Picogreen

for 10 minutes. Subsequently, the slides were sealed with an antifade solution (CAS#P0126-25ml, Beyotime, China) and visualized using a Laser Scanning Confocal Microscope (Zeiss, DEU).

Enzyme linked immunosorbent assay (ELISA)

The supernatant from cultured human HCC cells was collected for the detection of several cytokines, including human IL-18 (CAS# KHC0308, Proteintech, China), human IL-1β (CAS# KE00021, Proteintech, China), human IL-6 (CAS# EK106, Multi Sciences, China), human IFN-β1 (CAS# PA00063, Proteintech, China), and human DNASEL13 (CAS# EH14324, FineTest, China). Additionally, tumor tissue lapping liquid was analyzed for mouse IL-18 (CAS# EK218EG, Multi Sciences, China), mouse IL-1β (CAS# KE10003, Proteintech, China), mouse IL-6 (CAS# KE10091, Proteintech, China), and mouse IFN-β1 (CAS# EK2236, Multi Sciences, China). The ELISA kits were employed according to the manufacturer's instructions.

Quantitative real-time PCR analysis

In each well of a 6-well plate, 1×10^6 cells were seeded and treated according to the specified conditions. RNA was extracted from the cells using RNAiso Plus (CAS# 9109, TaKaRa, Japan), following the manufacturer's instructions. Subsequently, the RNA was reverse transcribed into cDNA using the PrimeScript RT Reagent Kit (CAS# RR047B (A × 4), TaKaRa, Japan). Quantitative PCR (qPCR) was conducted using a CFX384 system (Bio-Rad, USA) to assess the expression levels of target genes, with GAPDH serving as the internal control for PCR amplification. The $2^{-\Delta Ct}$ method was employed for the comparison and quantification of target gene expression. The primers utilized in this study are detailed in Supplementary Material Table S1.

Mitochondrial membrane potential detection

Mitochondrial membrane potential was assessed using a Mitochondrial Membrane Potential Detection Kit (CAS# C2005, Beyotime, China). Cells were incubated with JC-1 for 20 minutes and subsequently examined with a confocal microscope (Zeiss, DEU).

***In vivo* imaging system**

For *in vivo* imaging, mice harboring luciferase-positive liver cancer cells were administered VivoGlo Luciferin (CAS# P1043, Promega, USA) and positioned within a camera obscuration platform (Bruker, CH) following anesthesia. In a dark field, devoid of external light sources, the specific photons emitted by the mouse were captured.

Lentivirus transfection

Lentiviruses were utilized to package human DNASE1L3, the human DNASE1L3 R206C mutant, mouse Dnase1l3, as well as short hairpin RNAs (shRNAs) targeting human DNASE1L3, human AIM2, human STING1, and mouse AIM2, in addition to luciferase (Genechem, China). The specific sequences employed are detailed in Supplementary Material Table S2.
